# Independent Prognostic Factors for Predicting Wound Healing in People With Venous Leg Ulcers: A Systematic Review and Meta‐Analysis

**DOI:** 10.1111/wrr.70197

**Published:** 2026-08-17

**Authors:** Chunhu Shi, Joe Harvey, Sara Lloyd‐John, Zaid Hamoodi, Sonya Rafiq, Xinyue Zhan, Jo Dumville

**Affiliations:** ^1^ School of Health Sciences, Faculty of Biology, Medicine and Health, Manchester Academic Health Science Centre University of Manchester Manchester UK; ^2^ NIHR Applied Research Collaboration Greater Manchester (ARC‐GM) Manchester UK; ^3^ Blond McIndoe Laboratories, Division of Cell Matrix Biology and Regenerative Medicine, School of Biological Sciences, Faculty Biology, Medicine and Health University of Manchester Manchester UK; ^4^ NIHR Manchester Biomedical Research Centre, Manchester University NHS Foundation Trust, Manchester Academic Health Science Centre Manchester UK; ^5^ Division of Musculoskeletal and Dermatological Sciences, School of Biological Sciences The University of Manchester Manchester UK; ^6^ Upper Limb Unit, Wrightington Hospital, Wigan and Leigh Teaching Hospitals NHS Foundation Trust Wigan UK; ^7^ Centre for Epidemiology Versus Arthritis, Centre for Musculoskeletal Research, Division of Musculoskeletal and Dermatological Sciences University of Manchester, Manchester Academic Health Science Centre Manchester UK; ^8^ Centre for Biostatistics, School of Health Sciences, Faculty of Biology, Medicine and Health University of Manchester, Manchester Academic Health Science Centre Manchester UK

## Abstract

This first comprehensive systematic review aimed to summarise evidence on independent prognostic factors for wound healing in people with venous leg ulcers. In June 2025, we searched Ovid MEDLINE, Ovid Embase, CINAHL and additional resources for studies with 100 or more participants that used multivariable analyses and longitudinal data to explore the prognostic value of factors in this area. Two reviewers extracted data and assessed risk of bias using the Quality In Prognosis Studies (QUIPS) tool and certainty of evidence using the Grades of Recommendation Assessment, Development and Evaluation (GRADE) approach. We synthesised studies narratively and, where applicable, pooled data using meta‐analyses. We identified 30 studies with 66,099 participants living with venous leg ulcers. We rated 14 studies at moderate risk of bias, and the remaining 16 studies were at high risk of bias. The included studies explored 116 prognostic factors. The evidence is uncertain or limited for the majority of the 116 prognostic factors. There is moderate‐certainty evidence, suggesting no association between complete healing and the factors of age in years (HR 0.99, 95% CI 0.97 to 1.0; OR 1.00, 0.99 to 1.02, for every year increment), and the presence of total deep reflux in the leg veins (HR 0.89, 0.75 to 1.05). Those having the presence of dermatitis likely have lower odds of complete healing (OR 0.51, 0.50 to 0.52). Although a wide range of factors have been investigated as potentially associated with venous leg ulcer healing, the evidence base for most is uncertain or limited.

**Abbreviations**
CI: confidence interval
GRADE: Grades of Recommendation Assessment, Development and Evaluation (GRADE)
HR: hazard ratio
NHS: National Health Service
OR: odds ratio
PRISMA: Preferred Reporting Items for Systematic reviews and Meta‐Analyses
PROGRESS: PROGnosis REsearch Strategy
QUIPS: Quality In Prognosis Studies
RR: relative risk
SWiM: synthesis without meta‐analysis

## 1 Introduction

Venous leg ulcers are open wounds on the lower leg, largely caused by damaged or diseased leg veins [1]. Venous leg ulcers reduce people's health‐related quality of life: they cause pain, can be odorous, increase infection risk, and limit people's mobility and social activities [2].

Venous leg ulcers are the most common complex wounds in the United Kingdom [3] with point prevalence data estimating that, at any one time, approximately 20,000 people will have venous leg ulcers requiring National Health Service (NHS) care [3]. This care is mainly delivered by nurses in community settings, often via weekly appointments. The number of people with venous leg ulcers in conjunction with the large amount of nursing care they require means these wounds are costly to health services [4].

Research has found that people who have, or have had, complex wounds consider wound closure (being healed) to be their highest priority clinical outcome [5]. Data show that when people's venous leg ulcers are healed, their health‐related quality of life is measurably improved [6]. Healed wounds also require less resource use, thus saving individuals and health and care systems money. As venous leg ulcers often take months or years to heal with provision of good care, those affected want to understand how long healing might take [7]. It is also important for care services and health professionals to understand prognosis for education, patient management and broader service delivery considerations.

Prognosis (or prediction) research in health and care settings uses longitudinal data to help understand the risk of a future outcome (in this case wound healing) based on knowledge about a person and their condition [8]. A prognostic factor is any measure that, among people with a given health condition, is associated with a future clinical outcome [9]. Knowledge of prognostic factors helps identify those at high risk of a future outcome, allowing health professionals to make informed, personalised treatment decisions.

There are many different factors that can be associated with clinical outcomes in the field of wound care [10]. These can be at:

- The person level (e.g., sex and age but also things like psychosocial factors).
- The wound level (e.g., wound duration and size).
- The organisational and care level (e.g., receipt of evidence‐based, guideline recommended care and time to referral to appropriate care).
- The cellular level (e.g., protein biomarkers).

A plethora of different factors have been suggested as being prognostic for healing of venous leg ulcer. However, overarching appraisal and synthesis of this literature is limited. Our scoping exercise identified five relevant scoping or systematic reviews in this area. Four of them are reviews of individual factors: protease activity [11], cytokine factors [12], wound microbiome [13], and the percent of wound area change [14]. One scoping review summarised overarching evidence on the factors that have potential prognostic value for healing of common non‐traumatic skin ulcer types [10]. None of these reviews summarised comprehensive evidence on all potential prognostic factors for the complete healing of venous leg ulcers. Also, these reviews included suboptimal study designs (e.g., cross‐sectional studies), which were widely acknowledged to be limited for exploring the independent associations of potential factors and complete healing. We aimed to locate, appraise and summarise evidence on independent prognostic factors for wound healing in people with venous leg ulcers.

## 2 Materials and Methods

We follow the prognostic factor review guidance of the PROGnosis REsearch Strategy (PROGRESS) and Cochrane Prognosis Methods Group to conduct this systematic review [15]. We registered the review protocol on the International Prospective Register of Systematic Reviews (PROSPERO, CRD42024542023). We adhere to the Preferred Reporting Items for Systematic reviews and Meta‐Analyses (PRISMA) guideline [16].

### 2.1 Eligibility Criteria

We included reports of prospective and retrospective longitudinal studies that investigated any purported prognostic factor for venous leg ulcer healing. We included prospective data that have been collected for randomised controlled trials when these were clearly presented as longitudinal studies secondarily exploring prognosis and not as part of the exploration of effectiveness. We, however, did not specifically search for randomised controlled trials. To focus the findings of this review on the most relevant evidence, we included only studies that aimed to assess the prognostic value of factors while controlling for other factors, using a multivariable analysis (i.e., to identify factors that are independently prognostic when other potential confounding factors are adjusted for). To focus on the most useful and relevant data, we only included studies with 100 or more participants.

We included studies of people with a venous leg ulcer, managed in any care setting and receiving any type of treatment. We acknowledged that the methods for diagnosing venous ulceration varied; therefore, we accepted definitions used in each included study. We included studies in people with venous leg ulcers alongside people with other types of wounds (e.g., arterial ulcers, pressure ulcers, diabetic foot ulcers) provided the results for people with venous ulcers are presented separately, or if most participants (at least 75%) have leg ulcers of venous aetiology. Where wounds were described only as leg ulcers without information as to aetiology, we planned to assume that they were venous in origin because this is the most common type of leg ulcer [17]. However, we were able to verify and confirm that all included studies included venous leg ulcers.

We included studies that involved participants at any stage in their treatment pathway and recorded, where available, baseline data on the time since diagnosis and treatments previously given together with ongoing treatments. We included studies that involved participants with any infection status at baseline and recorded any available data on this. We excluded studies conducted solely in vitro and animal studies.

We acknowledge that there are two broad wound healing outcome domains: complete wound healing and change in wound size [18]. We included complete wound healing‐related outcomes as the primary outcomes:

- time to healing (analysed by survival analysis);
- proportion of people with ulcers completely healed, at any follow‐up duration. For binary outcomes we considered dichotomous summary data at the longest time‐point or the key time point specified in the study's methods section.

We accepted study authors' definitions of what constituted a healed wound. We did not consider outcomes related to changes in wound size or any variations of this measure—for example, wound size‐related outcomes describing hard‐to‐heal wounds or delayed wound healing—where wound closure was not the final event.

### 2.2 Study Identification

In June 2025, we searched the databases of Ovid MEDLINE (1946), Ovid Embase (1974) and CINAHL (1982). See Supporting Information for the search strategy used for Ovid MEDLINE. We developed search strategies based on the concepts of venous leg ulcer terms and prognosis filters [19, 20]. The venous ulcer terms were developed by the former Cochrane Wounds and had been widely used in Cochrane Reviews in this area. In addition, we searched the bibliographies of all included studies and of relevant systematic reviews we identified in this area [10, 11, 12, 13, 14] and others we located subsequently.

Two review authors independently assessed the titles and abstracts of retrieved records against the inclusion criteria with involvement of a third author as required. We obtained all potentially relevant studies in full, and two review authors independently assessed these for eligibility, again with involvement of a third reviewer as required.

When a single study was reported in multiple publications/reports, we obtained all publications. Each study was included only once in the review, but data were extracted from all relevant reports.

### 2.3 Data Extraction and Risk of Bias Assessment

We collected all association data reported between the prognostic factor and outcomes, with details on the adjustment factors used. Data were extracted using an Excel‐based data extraction sheet, which was piloted on a small number of studies. One review author extracted the data listed in Supporting Information, which were then checked by a second reviewer. Following data extraction, we organised related variables into broader groups when they were judged to represent the same underlying prognostic factor. For example, specific variables—area of the largest ulcer (cm^2^), wound size (cm^2^) and a binary indicator of wound area > 20 cm^2^—were grouped under the broader name of wound area. We grouped age‐related variables including age in years, and binary or categorical age indicators into the name of age. We then further grouped the prognostic factors into higher‐level categories: (1) patient‐level characteristics including demographics, socioeconomic factors, anthropometrics, comorbidities, psychological symptoms, lifestyle; (2) ulcer‐level characteristics including wound dimensions, physical status of wounds, wound location and number, wound condition assessment; (3) vascular‐related characteristics or related pathophysiology variables including arterial status, venous pathophysiology, venous history or condition; (4) characteristics related to calf muscle pump function such as mobility status, physical measurements; (5) medical care‐related factors including venous surgery, orthopaedic surgery; (6) surrogate indicators of healing such as wound area change; and (7) biomarkers and microbiology‐related factors. We developed this high‐level grouping based on findings from the scoping review by Jenkins and colleagues [10] on prognostic factors for healing of common skin ulcers. The grouping was used solely to structure reporting and did not inform the evidence synthesis.

Two review authors critically appraised the included studies using the recommended risk of bias tool for prognostic factor studies: Quality In Prognosis Studies (QUIPS) tool [21]. In the case of discrepancies, the review authors attempted to reach consensus; if necessary, a third review author resolved any disagreements. The QUIPS tool is specifically designed to assess prognostic factor review questions. It assesses study risk of bias based on domains of: study participation, study attrition, prognostic factor measurement, outcome measurement, adjustment for other prognostic factors and statistical analysis and reporting. Each domain was assessed based on reported information and judged as being at high, moderate or low risk of bias. We defined an all‐domain risk of bias per study for each prognostic factor–outcome combination, considering all the above domains. In line with the common recommendation [21], overall risk of bias was rated as low when all domains were judged to be at low risk; high when one or more domains were judged to be at high risk, or when all domains were unclear; and unclear when one or more domains were judged to be unclear but no domain was judged to be at high risk.

### 2.4 Data Synthesis and the Certainty of Evidence Assessment

We anticipated that results from multivariable analyses may be reported as odds ratios (OR), relative risks (RR) or hazard ratios (HR). We planned to use the ORs as the common measure of the association, using RRs and HRs to estimate ORs at a particular time point [22]. Once we extracted data, however, this was not possible because the information required for the conversion was not available (i.e., no reporting of the baseline risk of venous ulcer healing among control groups—those without specific prognostic factors). All adjusted estimates of associations were summarised within the broader prognostic factor groups and presented graphically. For example, evidence on the area of the largest ulcer (cm^2^), total wound area (cm^2^) and an indicator for wound area > 20 cm^2^ was organised under the prognostic factor “wound area”. However, when related variables were operationalised heterogeneously, meta‐analyses were performed separately by measurement type (e.g., ORs for the binary indicator were pooled separately from ORs for continuous measures).

We assessed the heterogeneity to determine if it was appropriate to undertake meta‐analyses of the prognostic association of a defined factor with venous leg ulcer healing. We considered the clinical heterogeneity of included studies based on the population, measure of the prognostic factor, outcome measurement and methodological heterogeneity due to study design/potential biases to make this final decision.

If data were available in more than two studies for a specific factor and we deem it appropriate, we performed meta‐analysis for this factor. We pooled data for all factors using random‐effects model. We summarised the meta‐analysis by the pooled estimate (the average prognostic factor effect), the Hartung–Knapp 95% confidence interval (CI) and the estimate of Tau^2^ (between‐study variance).

For factors where heterogeneity precludes meta‐analyses, we summarised data narratively and present the evidence following the Synthesis without meta‐analysis (SWiM) guidance [23]. We used the following three criteria to identify factors that show promise for future research—though not amenable to pooling: a statistically significant association with ulcer healing of *p* ≤ 0.01, a large magnitude of association (OR ≥ 2.0) and a sample size of 500 or more. This approach to select promising factors has been used previously [24].

Where only some of the available factor‐specific data from included studies were suitable for meta‐analysis and the others were not, we explored the consistency between meta‐analysis results and the related data that could not be pooled.

We used an approach modified from the Grades of Recommendation Assessment, Development and Evaluation (GRADE) framework to assess the certainty of the summarised evidence for each prognostic factor–outcome combination [25]. We rated the overall strength of evidence as high, moderate, low or very low considering the within‐study risk of bias, the directness of evidence, heterogeneity, precision of effect estimates and risk of publication bias [26]. We also considered the upgrading factors: large effect and dose effect, although we note that high risk of bias may artificially lead to large effects.

## 3 Results

**FIGURE 1:**
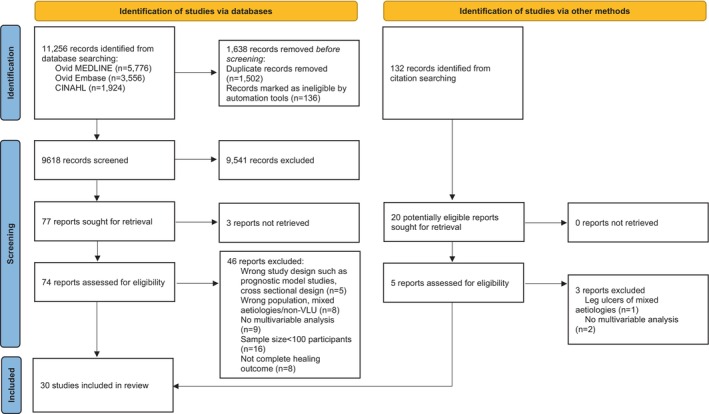
Flow diagram of the study selection.

We identified 11,256 records from database searches and an additional 132 from citation searches. We included a total of 30 studies in this review (see Figure 1) [27, 28, 29, 30, 31, 32, 33, 34, 35, 36, 37, 38, 39, 40, 41, 42, 43, 44, 45, 46, 47, 48, 49, 50, 51, 52, 53, 54, 55, 56].

### 3.1 Characteristics of the Included Studies

The 30 included studies were either prospective/retrospective cohort studies or cohorts derived from randomised controlled trials (see Supporting Information) [27, 28, 29, 30, 31, 32, 33, 34, 35, 36, 37, 38, 39, 40, 41, 42, 43, 44, 45, 46, 47, 48, 49, 50, 51, 52, 53, 54, 55, 56]. They had 66,099 participants, mostly 18 years or older, living with venous leg ulcers. The median of the reported study sample sizes was 259.5 (range: 100 to 29,189). The median study follow‐up duration across the 27 studies, where reported, was 24 weeks (range: 12 to 156). Of the 30 included studies, only 10 (33%) provided a clear definition of the complete healing outcome that is, 100% epithelialisation of the wound bed [28, 31, 32, 35, 38, 42, 43, 45, 46, 49]. Cox and logistic regression methods were each used in 12 studies, respectively.

The 30 studies explored 116 potentially prognostic factors. Among the higher‐level categories, patient‐ and ulcer‐related factors were the most reported in the included studies. Across the 27 studies with results reported, their multivariable analyses included a median of five factors (range: 1 to 34).

### 3.2 Risk of Bias Assessment Results

Of the 30 included studies, we rated 14 studies at moderate risk of bias (see Table 1) [27, 29, 34, 35, 38, 40, 42, 44, 45, 46, 48, 49, 53, 56]. The remaining 16 studies were judged to be at high risk of bias, with the selective reporting of analysis results as the most frequent limitations identified.

**TABLE 1:** Results of the risk of bias assessment.

	Study participation	Study attrition	Prognostic factor measurement	Outcome measurement	Study confounding	Statistical analysis and reporting	Overall judgement
Aloweni et al. [27]	Low	Moderate	Moderate	Moderate	Moderate	Low	Moderate
Barwell et al. [28]	Moderate	Moderate	Low	Moderate	Low	High	High
Cardinal et al. [29]	Moderate	Low	Moderate	Low	Moderate	Low	Moderate
Chaby et al. [30]	Moderate	Low	Moderate	Moderate	Low	High	High
Chan et al. [31]	Moderate	Low	Moderate	Low	High	Low	High
de Carvalho Bacelar et al. [32]	Low	High	Low	Low	Moderate	Low	High
Escaleira et al. [33]	Low	Moderate	High	Low	Moderate	High	High
Fife et al. [34]	Low	Moderate	Low	Low	Low	Low	Moderate
Finlayson et al. [35]	Moderate	Low	Low	Low	Low	Low	Moderate
Franks et al. [36]	Low	High	Low	Low	Moderate	Low	High
Gelfand et al. [37]	Low	Low	Moderate	Moderate	Moderate	High	High
Gohel et al. [38]	Moderate	Low	Moderate	Moderate	Low	Moderate	Moderate
Hart et al. [39]	Moderate	Moderate	Moderate	Moderate	Moderate	Moderate	High
Jull et al. [40]	Low	Low	Low	Low	Low	Low	Low
Karanikolic et al. [41]	Moderate	High	Moderate	Moderate	Low	Moderate	High
Kulkarni et al. [42]	Moderate	Low	Low	Moderate	Low	Moderate	Moderate
Lantis et al. [43]	Moderate	Moderate	Moderate	High	Moderate	Moderate	High
Margolis et al. [44]	Low	Moderate	Low	Low	Low	Low	Moderate
Marston et al. [45]	Moderate	Low	Low	Low	Low	Low	Moderate
Milic et al. [46]	Low	Moderate	Moderate	Moderate	Low	Low	Moderate
Moore et al. [47]	Moderate	High	Low	Moderate	High	High	High
Mościcka et al. [48]	Low	Moderate	Moderate	Moderate	Moderate	Moderate	Moderate
Parker et al. [49]	Low	Moderate	Low	Low	Moderate	Moderate	Moderate
Phillips et al. [50]	Moderate	Low	Moderate	Moderate	Low	High	High
Pihlaja et al. [51]	Low	Moderate	High	Moderate	High	Moderate	High
Rajhathy et al. [52]	Moderate	Moderate	Moderate	High	Moderate	Moderate	High
Skene et al. [53]	Low	Moderate	Moderate	Low	Moderate	Low	Moderate
Szewczyk et al. [54]	Moderate	Low	Moderate	Low	Moderate	High	High
Taylor et al. [55]	High	Low	Low	Moderate	Low	Moderate	High
Yang et al. [56]	Moderate	Moderate	Low	Moderate	Low	Moderate	Moderate

### 3.3 Findings of Evidence Syntheses on Independent Prognostic Factors

Among the 116 identified factors, we synthesised evidence, either quantitively or narratively, for only 32, as prognostic data for these were reported in two or more studies (see Table 2). See Supporting Information for details on the remaining 84 factors that were only reported in single studies.

**TABLE 2:** Summary of findings by specific prognostic factors.

Prognostic factor categories and specific factors	Definition	Outcome	No. of studies	No. of participants	Effect estimate (95% CI), random effects model	Heterogeneity	Certainty of evidence
Patient‐level characteristics							
Age	Age in years	Time to complete healing (median 24 weeks, range: 16 weeks to 3 years)	4	2202	HR 0.99 (0.97 to 1)	Tau^2^ < 0.0001 [0.0000; 0.0010]; *I* ^2^ = 50.0%	Moderate certainty
	Age in years	Complete healing (median 24 weeks, range: 12 weeks to 52 weeks)	6	2218	OR 1.00 (0.99 to 1.02)	Tau^2^ < 0.0001, *I* ^2^ = 22.2%	Moderate certainty
	Categories of age using different cut‐offs (3 studies) or results unspecified (1 study)	NR	4	1128	Narrative: Inconsistency with non‐significance (3/4 studies) suggesting no association, but significance (1/4 studies) suggesting better healing outcomes among older people	NR	NR
Female		Complete healing (12 weeks, 24 weeks)	2	1902	HR: 1.16 (0.13 to 10.32)	Tau^2^ = 0.0515; *I* ^2^ = 86.6%	Very low certainty
		Complete healing (12 weeks, 52 weeks)	4	966	OR: 0.63 (0.39 to 1.03)	Tau^2^ = 0; *I* ^2^ = 0	Low certainty
	Various definitions or reference unspecified	Complete healing (median 25 weeks, range 12 weeks to 3 years)	4	2248	Narrative: Consistency, no significance	NR	NR
Diabetes	Diabetes (yes/no)	Complete healing (median 24 weeks, range: 12 weeks to 3 years)	5	3077	HR: 1.04 (0.96 to 1.12)	Tau^2^ = 0; *I* ^2^ = 0	Low certainty
		Complete healing (median 24 weeks, range: 12 weeks to 52 weeks)	5	2029	OR: 0.99 (0.58 to 1.69)	Tau^2^ = 0.0609; *I* ^2^ = 23.5%	Low certainty
DVT	History or presence of DVT (yes/no)	Complete healing (median 24 weeks, range: 24 weeks to 27 weeks)	3	1673	HR: 0.85 (0.41 to 1.76)	Tau^2^ = 0; *I* ^2^ = 63.5%	Low certainty
		Complete healing (median 24 weeks, range: 12 weeks to 52 weeks)	7	2258	OR: 0.86 (0.67 to 1.11)	Tau^2^ = 0; *I* ^2^ = 0	Low certainty
Obesity	Obesity or BMI > 35 (yes/no)	Complete healing (52 weeks)	2	754	OR: 1.18 (0.09 to 15.44)	Tau^2^ = 0; *I* ^2^ = 0	Low certainty
Hypertension	Hypertension (yes/no)	Complete healing (median 24 weeks, range: 12 weeks to 52 weeks)	3	1152	OR: 0.99 (0.43 to 2.28)	Tau^2^ < 0.0001; *I* ^2^ = 0.0% [0.0%; 89.6%]	Low certainty
Coronary artery disease	Various conditions including coronary artery disease (1 study), myocardial infarction (1 study), cerebral vascular accident (1 study), angina (1 study)	Complete healing (24 weeks and 52 weeks)	2	814	Narrative: Inconsistency, largely the presence of conditions means poor healing	NR	Very low certainty
Peripheral arterial disease	Presence of peripheral arterial disease (yes/no)	Time to complete healing (6 months)	1	255	HR: 0.41 (0.13 to 1.27)	NR	Very low certainty
		Complete healing (median 24 weeks, range: 12 weeks to 52 weeks)	3	1272	OR: 0.74 (0.31 to 1.75)	Tau^2^ = 0; *I* ^2^ = 0%	Low certainty
Trauma	Presence of trauma (yes/no)	Complete healing (12 weeks, 52 weeks)	2	1331	OR: 1.03 (0.03 to 38.71)	Tau^2^ = 0.1043; *I* ^2^ = 62.9%	Very low certainty
Rheumatoid arthritis	Presence of rheumatoid arthritis (yes/no)	Complete healing (median 24 weeks, range: 24 weeks to 3 years)	3	2007	HR: 0.98 (0.57 to 1.69)	Tau^2^ = 0.0188; *I* ^2^ = 27.7%	Low certainty
		Complete healing (median 24 weeks, range: 24 weeks to 3 years)	2	1115	OR: 0.95 (0.29 to 3.17)	Tau^2^ = 0; *I* ^2^ = 0%	Very low certainty
Osteoarthritis	Presence of osteoarthritis (yes/no)	Complete healing (12 weeks)	2	1115	OR: 0.74 (0.46 to 1.20)	Tau^2^ = 0; *I* ^2^ = 0%	Very low certainty
Smoking	Smoking history (yes/no)	Time to complete healing (6 months)	1	255	HR: 0.94 (0.41 to 2.16)	NR	Very low certainty
		Complete healing (median 12 weeks; range: 12 weeks to 52 weeks)	2	1331	OR: 1.03 (0.55 to 1.95)	Tau^2^ = 0; *I* ^2^ = 0%	Low certainty
	Current smoker or unspecified (yes/no)	Complete healing (median 12 weeks; range: 12 weeks to 52 weeks)	2	1115	Narrative: Consistency in non‐significance, with one suggesting smoking increased healing but the other suggesting the opposite	NR	Very low certainty
Lipodermatosclerosis	Presence of lipodermatosclerosis (yes/no)	Complete healing (12 and 24 weeks)	2	598	OR: 1.26 (1.20 to 1.32)	Tau^2^ = 0; *I* ^2^ = 0%	Moderate certainty
Dermatitis	Presence of dermatitis (yes/no)	Complete healing (12 and 24 weeks)	2	598	OR: 0.51 (0.50 to 0.52)	Tau^2^ = 0; *I* ^2^ = 0%	Moderate certainty
BMI	BMI	Complete healing (or delayed healing), 12 weeks and 24 weeks	2	438	OR: 1.06 (0.23 to 4.87)	Tau^2^ = 0.0126; *I* ^2^ = 42.2%	Low certainty
Renal disease	Presence of renal disease (yes/no)	Complete healing (12 and 24 weeks)	2	877	OR: 1.18 (0.27 to 5.04)	Tau^2^ = 0; *I* ^2^ = 0.0%	Very low certainty
Ulcer‐level characteristics							
Wound duration	Wound duration (months)	Time to complete healing (median 24 weeks, range: 12 weeks to 3 years)	6	3166	HR: 0.99 (0.98 to 1.00)	Tau^2^ < 0.0001; *I* ^2^ = 85.9%	Low certainty
	Wound duration (months)	Complete healing (median 24 weeks, range 12 week to 12 months)	3	1152	OR: 0.88 (0.60 to 1.27)	Tau^2^ = 0.0160; *I* ^2^ = 80.4%	Low certainty
	Various definitions or references, ulcer chronicity (1 study), wound duration in months on a log scale (2 studies), wound duration > 12 months (1 study), definition or results unspecified (3 studied)	Complete healing (median 16 weeks, range 12 weeks to 3 years)	7	57,284	Narrative: Significance (5/7 studies), non‐significance or unclear (2/7 studies); all consistently suggesting that shorter wound duration, better healing outcomes	NR	NR
Wound area	Size cm^2^	Complete healing (median 9 months, range: 12 weeks to 3 years)	5	1979	HR: 0.96 (0.91 to 1.01)	Tau^2^ = 0.0017; *I* ^2^ = 93.0%	Very low certainty
	Size cm^2^	Complete healing (median 12 weeks, range: 12 weeks to 12 months)	4	1929	OR: 0.90 (0.76 to 1.08)	Tau^2^ = 0.0115, *I* ^2^ = 93.0%	Very low certainty
	Various definitions or references, wound area change percentage or < 25% at week two (2 studies), wound area on a log_10_ scale (2 studies), wound area > 20 cm^2^ (1 study), unspecified definition or no result (3 studies)	Complete healing (median 24 weeks, range: 12 weeks to 52 weeks)	8	84,514	Narrative: Significance (4/5 studies), non‐significance (1/5 studies); all consistently suggesting that smaller areas, better healing outcomes	NR	NR
Infection	Presence of wound infection (yes/no)	Complete healing (median 24 weeks, range: 12 weeks to 24 weeks)	3	27,068	Narrative: Conflicting results: significance (2/3 studies), suggesting that infection delayed the complete healing of venous leg ulcers; non‐significance (1/3 studies)	NR	Low certainty
Wound covered with fibrin > 50%	Yes/no	Complete healing (24 and 52 weeks)	3	560	Narrative: Heterogenous and inconsistent results among the three studies, with one significant result and a non‐significant result suggesting that > 50% wounds with fibrin delayed healing but the other with non‐significance suggesting the opposite	NR	Very low certainty
No. of limbs	Two limbs involved (yes/no)	Complete healing (12 weeks, 6 months)	2	548	OR: 1.08 (0.86 to 1.37)	Tau^2^ = 0; *I* ^2^ = 0	Low certainty
Characteristics related to calf muscle pump function							
Mobility	Various definitions or references, wheelchair/bedbound (*n* = 1 study), some problem walking (1 study), daily walking distance < 200 m (1 study), unable to walk 1 block (1 study), leg mobility vs. immobile (1 study), unspecified (1 study)	Complete healing (median 18 weeks, range: 12 weeks to 3 years)	6	2421	Narrative: No significance (4/6 studies); significance (2/6 studies); all favouring the categories of no mobility problem regardless of the definitions used	NR	Low certainty
Vascular‐related characteristics or related pathophysiology variables							
ABPI	ABPI < 0.8 (yes/no)	Complete healing (24 weeks)	2	360	Narrative: Inconsistency and conflicting results, with one suggesting ABPI < 0.8 delayed healing but the other suggesting ABPI < 0.8 predicted healing	NR	Very low certainty
	ABPI values	Complete healing (median 24 weeks; range 12 weeks to 24 weeks)	3	698	OR: 0.38 (0.04 to 3.50)	Tau^2^ = 0; *I* ^2^ = 0%	Low certainty
Small saphenous vein reflux	Yes/no	Complete healing (24 weeks)	2	514	Narrative: Consistency, no significance, with one favouring the factor but the other not	NR	Very low certainty
Segmental deep reflux	Yes/no	Complete healing (24 and 30 weeks)	2	1421	HR: 1.01 (0.69 to 1.47)	Tau^2^ = 0; *I* ^2^ = 0.0%	Moderate certainty
Total deep reflux	Yes/no	Complete healing (24 weeks)	2	1422	HR: 0.89 (0.75 to 1.05)	Tau^2^ = 0; *I* ^2^ = 0.0%	Moderate certainty
Superficial venous reflux	Yes/no	Complete healing (24 weeks and 3 years)	2	1773	HR: 1.14 (0.85 to 1.53)	Tau^2^ = 0; *I* ^2^ = 0.0%	Low certainty
		Complete healing (unspecified timepoint)	1	259	OR: 0.12 (0.01 to 0.30)	NR	Low certainty
Incompetent perforators	Yes/no	Complete healing (24 weeks)	2	1423	HR: 1.04 (0.48 to 2.28)	Tau^2^ = 0; *I* ^2^ = 0%	Low certainty
Varicose veins	Yes/no	Complete healing (median 12 weeks; range 12 to 24 weeks)	3	1375	OR: 1.16 (0.41 to 3.28)	Tau^2^ = 0.1182; *I* ^2^ = 37.6%	Very low certainty
Medical treatment‐related variables							
Debridement	Yes/no	Complete healing (median 24 weeks; range 24 weeks to 52 weeks)	3	840	OR: 0.44 (0.17 to 1.10)	Tau^2^ = 0; *I* ^2^ = 0%	Moderate certainty
Compression	Various definitions including compression pressure (2 studies), compression duration in days (1 study)	Complete healing (24 weeks)	3	874	Narrative: All significance; with consistency in suggesting compression improved healing	NR	Moderate certainty
Veins surgery	Yes/no	Complete healing (median 24 weeks, range 24 weeks to 3 years)	3	2011	HR: 0.99 (0.80 to 1.23)	Tau^2^ = 0; *I* ^2^ = 0%	Moderate certainty
		Complete healing (median 24 weeks, range 12 weeks to 24 weeks)	3	1141	OR: 1.11 (0.01 to 131.05)	Tau^2^ = 3.2571; *I* ^2^ = 86.7%	Very low certainty

There is *moderate‐certainty evidence* on the association between complete healing and five factors but low or very low‐certainty evidence for the remaining 27 factors. *Moderate‐certainty* evidence suggests no prognostic association between any measures of complete wound healing outcomes and age in years (4 studies, 2202 participants, HR 0.99, 95% CI 0.97 to 1.0; 6 studies, 2218 participants, OR 1.00, 95% CI 0.99 to 1.02, for every year increment). Those having the presence of dermatitis likely have lower odds of complete healing (2 studies, 598 participants, OR 0.51, 95% CI 0.50 to 0.52; *moderate‐certainty evidence*). The presence of total deep reflux may reduce the likelihood of complete healing, but there is uncertainty and the findings are not statistically significant (2 studies, 1422 participants, HR 0.89, 95% CI 0.75 to 1.05; *moderate‐certainty evidence*). The included prognostic studies also assessed the risk of healing for two treatment‐related variables: the use of the ulcer debridement and compression therapies (Supporting Information). Their results were not interpreted in this review as the related treatment effectiveness should be assessed in randomised controlled trials.

Evidence is either *low‐ or very low‐certainty* for the remaining 27 potentially prognostic factors that we synthesised evidence for (see Table 2), with inconsistency and imprecision the most common reasons for downgrading the evidence certainty. The low or very low certainty of the evidence indicates substantial uncertainty regarding whether the observed findings in Table 2 reflect the true prognostic associations, and the uncertainty should not be interpreted as evidence of no association between the potential factors and complete healing. Thus, we are unclear whether these specific factors are prognostic for the complete healing of venous leg ulcers, and they include:

- 14 patient‐level characteristics: sex, BMI, presence of diabetes, history of deep vein thrombosis, obesity, presence of hypertension, presence of coronary artery diseases, presence of peripheral arterial disease, history of trauma to lower limb(s), presence of rheumatoid arthritis, presence of osteoarthritis, current or history of smoking, presence of renal diseases and the presence of lipodermatosclerosis;
- five wound‐level characteristics: wound duration, wound area, presence of wound infection, the number of limbs involved, presence of wound covered with fibrin > 50%;
- six vascular‐related characteristics or pathophysiology variables: ABPI < 0.8, presence of small saphenous vein reflux, presence of superficial venous reflux, presence of segmental deep reflux, presence of incompetent perforators, presence of varicose veins;
- one calf muscle pump function‐related characteristic: impaired mobility, and
- one medical treatment‐related variable: history of veins‐related surgery.

Among the 84 factors reported in single studies only (Supporting Information), the Margolis index score (categorised as 0, 1 and 2) reported a large magnitude of association with the complete healing of venous leg ulcers (OR ≤ 0.5) and a large sample size (*n* = 802) and from a study at low risk of bias [40]. The scoring system is based on two wound‐level prognostic factors—ulcer area and duration in months—to classify venous leg ulcers into three risk categories to identify hard‐to‐heal wounds [40].

## 4 Discussion

Current research gives us relatively little insight into evidence on independent prognostic factors for venous leg ulcer healing. We found 30 studies (*n* = 66,099 participants) meeting our criteria of using appropriately designed longitudinal studies to explore potential associations between prognostic factors and complete wound healing of venous leg ulcers. For most of the 116 potentially variables identified, evidence for their associations with complete wound healing were uncertain or limited. We found moderate‐certainty evidence suggesting that the likelihood of complete healing did not change with increasing age (in these models where other factors were adjusted for), a finding that appears to contrast with anecdotal views regarding the role of age in wound healing. One of the possible explanations is that older age may act as a ‘proxy’ for other potential prognostic characteristics (e.g., co‐morbidities) and may correlate with, or confound, other potentially independent factors included in multivariable analyses. Also, the presence of total deep reflux in the leg veins may not be prognostic for venous leg ulcer healing. Our low‐ or very low‐certainty ratings for most factors should not be interpreted as implying that all of the previously reported associations in the related studies are invalid, or that the factors are not prognostic of complete healing. Rather, the certainty of evidence indicates the extent to which the whole body of the available evidence that met our eligibility criteria supports confident conclusions about independent prognostic value after considering risk of bias, inconsistency, imprecision and publication bias in the included studies.

### 4.1 Comparisons With Other Studies

Our scoping identified five reviews that explored prognostic factors for wound healing, including venous leg ulcer populations [10, 11, 12, 13, 14]. Three of these reviews included data from univariate analysis and studies of suboptimal designs for exploring prognosis, such as cross‐sectional studies. Thus, the independent associations of potential prognostic factors and complete healing were not rigorously explored. Exceptions were Norman and colleagues' systematic review of prognosis research on whether aspects of the wound microbiome are independently prognostic for wound healing in people with complex wounds including venous leg ulcers [13]. One cohort study of people with venous leg ulcers (228 participants) from Norman and colleagues was also included in this current review [43]. Westby and colleagues summarised prognosis evidence from 11 cohort studies, aiming to explore whether protease activity is an independent prognostic factor for the healing of venous leg ulcers [11]; however, none of the included studies reported multivariable analysis data. Thus, no protease‐related research was identified and included for the present review. All but one of the existing reviews focused on exploring prognostic associations for one factor, thus having a narrower focus than the current work. For example, Burian and colleagues reviewed 28 studies (790 participants) to investigate the association of cytokine factors (38 different factors in total) with venous leg ulcer healing [12]. Gwilym and colleagues reviewed evidence on using percent area reduction as a predictor of complete healing of diabetic foot ulcers and venous leg ulcers [14]: among the included studies only five involved people with venous leg ulcers. The scoping review by Jenkins and colleagues summarised evidence on prognostic factors associated with wound healing for various types of common non‐traumatic skin ulcers [10]. The review provided no evidence on prognostic associations of specific factor and did not report critical appraisal of the evidence identified [10].

We found, as did the broader review, that it is uncertain whether there is a prognostic association between wound infection and wound area and complete wound healing, in the case of this review—this was for venous leg ulcers specifically [13, 14]. For the other factors noted in existing reviews such as cytokine factors and protease activity, our current review did not identify sufficient data from multivariable analysis for synthesis. Further research may be required to explore their independent value for predicting the complete healing of venous leg ulcers.

### 4.2 Implications for Practice and Research

Suggested prognostic factors for venous leg ulcer healing are prevalent in the research literature—but often the studies generating underlying evidence are not robust, being of ill‐suited design and leading to high risk of bias. This review suggests that decision makers and researchers need to exercise caution when assuming prognostic associations with wound healing based on long‐held assumptions, such as with age. The variable of age is widely considered as confounders in various fields, and it might confound a potential relationship between other potential factors (e.g., co‐morbidities) and venous leg ulcer healing. Prognostic associations between potential factors and complete healing can be complex and challenging to explore, thus requiring careful data collection and the use of appropriate statistical analysis (e.g., multivariable models) to isolate independent associations accounting for confounders [9]. For most potential factors, based on research evidence, we are uncertain about their prognostic associations. As we note in the introduction to this work, prognosis data are important for health professionals, patients and their families and those who plan care delivery. A thorough understanding of prognosis data is also important for planning robust research. Currently, we lack optimal evidence‐informed insights to target people whose ulcers may be slow to heal, and even with evidence‐based treatments, to identify those who likely to experience delayed healing. The evidence uncertainty is also a barrier to the shared decision‐making where patient preference should be considered in choosing treatments.

According to the GRADE approach, the low or very low‐certainty rating of the evidence for most factors was mainly due to serious or very serious study limitations, and the imprecision of the estimate. These limitations are likely related to the prominence of exploratory studies with small sample sizes and suboptimal methods—two commonly observed and widely acknowledged issues in prognosis research [57, 58]. The median sample size in this review (*n* = 259.5) may be insufficient to yield precise estimates from multivariable analyses, particularly given that half the studies included at least five prognostic factors. Some cases of our evidence syntheses were downgraded for unexplained heterogeneity and the presence of publication bias. Under‐reporting of regression coefficients for all factors, particularly ‘non‐significant’ factors, is prevalent in prognosis research. This issue means that meta‐analyses in prognosis fields may likely involve incomplete data and yield potentially biased estimates [57]. To address these methodological issues on this topic, future research should undertake cohort studies following the best practices and established methods, enrolling sufficient participants and reporting the full results of regression analysis. In terms of the sample size calculation, the rule of thumbs is to ensure more than 10 events per variable. Despite no related guide for prognostic factor research, Jinks and colleagues and Riley and colleagues have proposed methods for calculating sample sizes for multivariable prognostic modelling research [59, 60]. Future research should be reported in compliance with reporting guidelines for observational studies, such as the REporting recommendations for tumour MARKer prognostic studies checklist, so as to completely present not only factors with ‘significant’ results but also those with non‐significant modelling results [61]. In addition, future research should clearly define the prognostic factors explored and the complete healing outcomes. In this systematic review, under‐reporting of factor definitions, measurement methods and where relevant the level of variable categories in the included studies hindered the appropriate grouping of related variables across the studies. Using the criteria of Wang and colleagues [24], we identified the Margolis index score as a promising factor for future research. However, this scoring system is based on the two wound‐level characteristics: ulcer area and duration in months [40], and the evidence base is uncertain for both characteristics. Future research may be able to focus on them.

### 4.3 Strengths and Limitations

In this current review, we used the well‐established methods for conducting prognostic factor reviews including the use of the validated Quality In Prognosis Studies tool to assess risk of bias, and the use of the GRADE approach to facilitate the appropriate interpretation of our meta‐analysis results. Whereas none of the previous reviews followed these methods. The other strength of this review is the comprehensiveness of its literature searches with the use of validated prognosis study search filters.

This review has some limitations. First, due to the small number of the included studies for each meta‐analysis, we were unable to explore the cause of unexplained heterogeneity that is very common in prognosis reviews in other fields [57]. In prognosis research, many issues can cause heterogeneity, for example, variations in settings and populations, adjustment for different sets of factors, the use of different methods to assess factors and outcomes, and different follow‐up durations [57].

Second, we did not contact authors to collect unpublished results for factors that might have been relevant to the review. When we made efforts to contact authors for a previous work on wound care [62], we were only able to collect very limited data that were not published by the original authors and the inclusion of such data did not change the findings. Schmucker and colleagues conducted a methodological review on whether the inclusion of unpublished data changes pooled effect estimates in meta‐analyses and interpretation. They found that only 3 out of 41 meta‐analyses resulted in different results [63].

Thirdly, we acknowledge a potential risk of selection bias in this review. Although we applied two well‐established and validated search filters for prognosis research in this review [19, 20], the identification of prediction studies remains challenging. Unlike randomised trials, prediction and prognosis studies are not consistently indexed in bibliographic databases [19], and there is currently no robust approach to ensure comprehensive retrieval in prognosis reviews. Because of these reasons, potentially relevant studies may have been missed, but it is challenging to explore to what extent they were missed.

In addition, some clinical trials include multivariable analyses to adjust for confounding when estimating treatment effects, for example, Gohel et al. [64]. However, these studies are not reliably captured by prognosis‐specific search filters, which often do not include trial‐related search terms, potentially further contributing to selection bias.

We also recognise that prognostic factors of interest may often be non‐modifiable (e.g., age) and thus not subject to experimental control; therefore, observational study designs are generally considered the most appropriate source of evidence for prognosis research [65]. While multivariable analyses conducted within trials may identify associations between variables and outcomes, their primary purpose is to estimate treatment effects. As such, these associations suggest whether the included variables are *predictive* of treatment effect [66] and may not necessarily represent true *prognostic* relationships independent of treatment. Predictive factors, which are associated with differential treatment effects and can be explored through comparisons between treatment groups in randomised trials, should be distinguished from prognostic factors, which reflect the natural history of a condition [67]. When randomised trial data are used, prognostic factors should ideally be examined within a single treatment group alone, rather than through comparisons between trial group [65]. Thus, while we did include analyses from trials when they were clearly presented as longitudinal studies secondarily exploring prognosis (and not as part of the exploration of effectiveness), other potentially prognostic insights from trial data are not represented here. Further and cautious exploration could be considered in individual prognostic factor‐focused reviews to complement this broad review, which raises important insights and questions about prognostic evidence on venous leg ulcer healing.

## 5 Conclusions

Although an extensive body of research has investigated a broad array of factors potentially associated with venous leg ulcer healing, the evidence is uncertain or limited for most of the factors identified. More studies are required; research can use the well‐established prognosis research methods using appropriate longitudinal designs and multivariable analysis.

## Conflicts of Interest

The authors declare no conflicts of interest.

## Supporting information


**Table S1:** Characteristics of the included studies.
**Table S2:** Results of data synthesis for all the 117 factors alongside GRADE assessment results where applicable.

## Data Availability

The data that support the findings of this study are available in the Supporting Information of this article.
