# Supplementary material for: Independent Prognostic Factors for Predicting Wound Healing in People With Venous Leg Ulcers: A Systematic Review and Meta‐Analysis
Source: Wound Repair Regen. 2026 Aug 17;34(4):e70197. doi: 10.1111/wrr.70197 (PMC13479044; doi:10.1111/wrr.70197)
Supplement: Supplementary file 1 [file WRR-34-0-s001.docx]

**Supporting Information**

**(1) Search strategy used for the Ovid Medline**

1	Stratification.ab,kw,ti.

2	exp ROC Curve/

3	Discrimination.ab,kw,ti.

4	Discriminate.ab,kw,ti.

5	c-statistic.ab,kw,ti.

6	c statistic.ab,kw,ti.

7	Area under the curve.ab,kw,ti.

8	AUC.ab,kw,ti.

9	Calibration.ab,kw,ti.

10	Indices.ab,kw,ti.

11	Algorithm.ab,kw,ti.

12	Multivariable.ab,kw,ti.

13	1 or 2 or 3 or 4 or 5 or 6 or 7 or 8 or 9 or 10 or 11 or 12

14	Validat*.af.

15	Predict*.ti.

16	Rule*.af.

17	Predict*.af.

18	Outcome*.af.

19	Risk*.af.

20	Model*.af.

21	18 or 19 or 20

22	17 and 21

23	History.af.

24	Variable*.af.

25	Criteria.af.

26	Scor*.af.

27	Characteristic*.af.

28	Finding*.af.

29	Factor*.af.

30	Decision*.af.

31	Identif*.af.

32	Prognos*.af.

33	17 or 20 or 30 or 31 or 32

34	23 or 24 or 25 or 26 or 27 or 28 or 29

35	33 and 34

36	Clinical*.af.

37	Logistic Models/

38	20 or 36 or 37

39	30 and 38

40	Prognostic.af.

41	20 or 34

42	40 and 41

43	14 or 15 or 16 or 22 or 35 or 39 or 42

44	13 or 43

45	exp Leg Ulcer/

46	(varicose ulcer* or venous ulcer* or leg ulcer* or stasis ulcer* or (lower extremit* adj ulcer*) or crural ulcer* or ulcus cruris or ulcer* cruris).tw.

47	45 or 46

48	44 and 47

49	exp animals/ not humans.sh.

50	48 not 49

**(2) Data extraction items**

We extracted the following data items using an Excel-based data extraction sheet:

- country and setting in which study was conducted;
- study design;
- eligibility criteria;
- participant details;
- ulcer details, including duration of ulcer, ulcer size, number of ulcers and ulcer history, and ulcer severity (with the system used to classify this) as reported by the study authors;
- treatment details, including compression and debridement (including type and frequency);
- details of each prognostic factor. As part of the extraction, potential prognostic factors identified in the included studies were grouped by reviewers and cross checked amongst the team when the factors were not specified clearly.
- details of each outcome (as detailed in outcome section above)
- duration of follow-up;
- type of analysis: e.g. logistic regression, Cox regression;
- any adjustment factors considered in the analysis;
- association statistics for each prognostic factor and healing outcomes e.g., odds ratios (ORs), hazard ratios (HRs) and their Confidence Intervals (CIs), variances, standard errors;
- loss to follow-up and reasons.

**Table 1. Characteristics of the included studies**

| Study ID | Countries of conduct | Study design | Study population | Number of participants recruited | Duration of follow-up | Healing outcome (as reported in studies) | Analytical approach (as described in paper) | Prognostic factors reported (the number of factors with multivariable analysis data reported; but the factors adjusted for but without results are not listed) |
| --- | --- | --- | --- | --- | --- | --- | --- | --- |
| Aloweni 2022 | Singapore | Retrospective cohort | Retrieved and reviewed electronic medical records of patients diagnosed with a VLU according to the International Statistical Classification of Diseases and Related Health Problems (ICD)‑10 code | 380 participants | 3 and 6 months | Healing (yes / no) at 3 months and 6 months | Multivariate logistic regression | 6 factors:  Age  Duration of compression therapy  Race  Peripheral vascular disease  Debridement surgery done  Both limbs affected with venous leg ulcer |
| Barwell 2000 | UK | Prospective cohort | Community based leg ulcer service. Five clinics in East Gloucestershire in 1995 referred all patients to one-stop vascular assessment clinic at Cheltenham General Hospital. ABPI >0.85. | 633 limbs from 587 participants | 3 years after ulcer healing | Complete ulcer healing and recurrence rates. Healing defined as complete re-epithelialization of the wound | Cox regression model | 3 factors:  Age  Popliteal vein reflux  Ulcer chronicity |
| Cardinal 2009 | United States | Prospective cohort (derived from a trial) | Patients with chronic venous ulceration | 338 patients analysed | 24 weeks | 100% ulcer closure (week 12) | Logistic regression | 34 factors:  Ulcer position (ankle:calf), shape change over time, initial wound area (cm2), wound duration (weeks), swelling (yes : no), initial wound shape, sex (M vs F), height (cm), weight (kg), BMI, age (years), ABPI, anaemia (yes : no), diabetes, stroke, hypertension, PVD, congestive heart failure, rheumatoid arthritis, osteoarthritis, alcohol consumption, smoking, DVT, recurring ulcer, bilateral ulcer, hyperpigmentation, varicosities, lipodermatosclerosis, dermatitis, leg mobility (mobile vs immobile), wound exudate (less vs more), necrotic tissue (%), pain (less vs more) |
| Chaby 2013 | France | Prospective cohort | Ambulatory or hospitalized patients, >50 years old, with more than one confirmed VLU lasting ≥ 1 month and with wound area > 1 cm2. Established venous disease confirmed by venous doppler duplex ultrasonography, ABPI ≥ 0.8. | 104 ulcers from104 participants | 24 weeks | Complete healing at week 24, without further definition | Multivariate models | 2 factors:  Surgery for superficial venous insufficiency during follow-up  Percentage of the reference VLU (i.e. the largest VLU) area reduction at W4 |
| Chan 2023 | Singapore | Prospective cohort | Patients ≥ 21 years and above with clinically diagnosed VLUs | 255 participants | 6 months | Wound healing (incidence of complete wound healing and time to wound healing), defined complete epithelization of VLU | Cox regression | 13 factors:  Age  Ethnicity  Obesity  Smoking history  Peripheral arterial disease  Prior wound history  History of DVT  Great saphenous vein reflux  Small saphenous vein reflux  Size of wound at first encounter  Duration of wound at start of study  Presence of slough in wound  Presence of wound infection |
| de Carvalho Bacelar 2025 | Brazil | Retrospective cohort (registry data) | Patients with venous leg ulcers selected from the state public reference center for vascular and advanced venous diseases and treated with ultrasound guided foam sclerotherapy | 336 legs from 279 patients | 52 weeks | Complete epithelialisation of the wound | Cox regression | Not reported |
| Escaleira 2010 | Portugal | Prospective cohort | Patients with VLUs recruited from one centre. ABPI needed to be ≥0.8 | 193 participants | 12 months or until their ulcers healed | Time to wound healing: determined from the difference between baseline and the day that complete healing was diagnosed | Cox proportional hazards model | 2 factors:  Wound area  Wound duration |
| Fife 2020 | USA | Retrospective cohort | Data from electronic health records from the Wound Registry Database. | 26713 ulcers | Each VLU had at least two clinical encounters; there were at least 5 days between first and last encounter; the gap between two clinic visits was not more than 90 days | Hierarchical definition of a wound considered healed using the wound Healing Index. | Multivariable logistic regression model. | 6 factors:  Wound area  Wound duration  Mobility of patients at arrival  Number of ulcers or wounds of any type  Infection  Patient admitted for acute hospital stay or emergency department visit |
| Finlayson 2017 | Australia | Secondary analyses of four primary cohort studies *(add reference for these)* | People with ulcer of venous aetiology (primarily), recruited wound clinics, hospital outpatient or community nursing services. ABPI needed to be between 0.8 and 1.2. If multiple ulcers the largest was selected | 247 participants | 24 weeks | Time ulcer being healed. A healed wound defined as complete epithelialization,  maintained for at least two weeks. | Cox proportional hazards model | 8 factors:  Age  Wound area  Wound duration  Sex  Mobility  High level compression therapy  PUSH score  Pain, depression, fatigue,  and sleep disturbance (PDFS) symptoms cluster |
| Franks 1995 | United Kingdom | Prospective cohort | Patients with VLU recruited from community leg ulcer clinics | 168 participants | 12 weeks | Number of ulcers healed (within 12 weeks) | Logistic regression model | 9 factors with multivariable analysis results available:  Age  Marital status  Social class  Employment status  Income  Social security benefits  Housing type  Level of Social isolation  Number of affected limbs |
| Gelfand 2002 | United States | Retrospective cohort (registry data) | Patients with venous leg ulcers of < 2 cm depth, size < 150 cm2 | 29,189 patients | 24 weeks | Complete healing | Logistic regression | 2 factors:  Initial wound log area, wound log duration |
| Gohel 2005 | UK | Retrospective cohort | Patients with open or recently healed leg ulceration of >4 weeks duration (CEAP grades C5 and C6) and ABPI >0.85 | 1324 legs from 1186 consecutive patients | 24 weeks | Ulcer healing, defined as complete re-epithelialisation of the wound | Cox regression proportional hazards model | 12 factors:  Age  Male sex  Right leg  History of deep venous thrombosis  Diabetes mellitus  Rheumatoid arthritis  Ulcer chronicity  Segmental deep reflux  Total deep reflux  Presence of incompetent calf perforating veins  Superficial venous reflux—no surgery  Superficial venous reflux—treated with surgery |
| Hart 2025 | United States | Longitudinal data, using an unspecified design | Patients with an active VLU presenting to an urban medical centre | 354 participants | 24 weeks | Complete healing, without specifying definition | Multivariable analysis, without specifying the method | 1 factor:  Moderate and severe Prognostic Nutritional Index (PNI) |
| Jull 2023 | New Zealand | Post-hoc secondary analysis of four RCTs | Recruited from community nursing services, ≥18, be able to provide informed consent, meet clinical case definition for VLU (ulcer present for at least 4-6 weeks, previous history of VLU, clinical indications for VLU, ABPI over 0.8 in 2 trials and over 0.7 in two trials, tolerant of compression for at least one week and other causative aetiology ruled out), as well as other criteria regarding specific intervention for each study | 802 participants | 12 weeks (2 trials), 24 weeks (2 trials) | Time to complete healing and proportion healed at either 12 or 24 week trial endpoints | Cox proportional hazard regression controlling for baseline confounders and the cluster effect of the trial | 1 factor:  Margolis index score |
| Karanikolic 2015 | Serbia | Prospective cohort | Venous ulcerations of the lower extremities | 100 participants | 24 weeks | Delay of healing; not fully described (likely not healing by 24 weeks according to results) | Binary logistic regression | 9 factors:  Age  Sex  BMI  Waist/hip ratio  ABPI  ABPI<0.08  Diabetes mellitus  Deep vein thrombosis  Chronic renal failure |
| Kulkarni 2007 | UK | Prospective cohort (Prospective baseline study and secondary prospective study) | Recruited from leg ulcer service, open leg ulceration for more than 4 weeks duration, ABPI of 0.85 or more and superficial venous reflux alone or mixed superficial and deep venous reflux on duplex imaging. | 229 participants in baseline study and 86 participants in secondary study | 24 weeks | Ulcer healing at 24 weeks. Healing defined as complete re-epithelialization of the wound and all healing dates were noted | Cox proportional hazards model | 12 factors:  Age  Wound duration  Sex  Ulcer location  History of deep vein thrombosis  Diabetes mellitus  Rheumatoid arthritis  Segmental deep reflux  Total deep reflux  Incompetent perforators  Superficial venous surgery  Venous refill time |
| Lantis 2013 | North America | Randomised controlled trial | Patients with duplex-confirmed venous disease and a VLU of 6-104 weeks’ duration and 2-12 cm 2 area (the maximal area covered with one spray of HP802-247) prior to debridement. | 228 participants | 12 weeks | Complete wound closure, defined as re-epithelialization, absence of drainage, and lack of need for a wound dressing persisting for 2 weeks | Multivariate logistic regression | 2 factors:  Wound area  Wound duration |
| Margolis 1999 | USA | Retrospective cohort | Study performed using histories from patients with chronic venous leg ulcers who were treated in the Cutaneous Ulcer Centre of the Department of Dermatology of the University of Pennsylvania Medical Center, Philidelphia, from 1993 through 1995. | 260 participants | Must have visited twice within 2 months of initial visit | For a wound to be considered healed, it must have healed within 24 weeks after the initiation of care. | Multivariable logistic regression model | 6 factors:  Wound area  Wound duration  Venous ligation or stripping  Hip or knee replacement surgery  >50% of wound covered with fibrin  ABPI <0.8 |
| Marston 2017 | USA, Canada, Germany, Poland, Hungary, Czech republic and Belgium | Retrospective cohort (retrospective analysis of data from three prospective randomised trials analysed as cohort studies) | Patients with venous reflux confirmed by Doppler ultrasound, a VLU located between the knee and above malleolus. Ulcers had to be present for at least 6 weeks but not more than 104 weeks, ABPI at least 0.8. | 716 participants | 12-16 weeks | Time to wound closure: defined as re-epithelialisation without drainage by the end-of-treatment time point (12 or 16 weeks) | Cox proportional hazards model | 4 factors:  Wound area  Wound duration  Gender  Diabetes |
| Milic 2009 | Serbia | Prospective cohort | Patients ≥ 16 years with leg ulceration of venous etiology, ulcer surface >5cm^2^, duration >3 months | 200 participants | 52 weeks | Complete ulcer healing, defined as the point at which complete epithelialization of the reference limb occurred. If a new ulcer appeared on the same limb after original ulcer closure this limb was considered to be open until the new ulcer closed. | Multivariate logistic regression using unconditional enter method | 15 factors:  Age  Wound area  Wound duration  Sex  Previous operations  Deep vein thrombosis  BMI  Reduction in calf circumference  Daily walking distance  Calf:ankle circumeference ratio  Fixed ankle joint ROM  Wound debrided surgically  Wound covered with fibrin  Depth of the wound  Emergence of new skin islets |
| Moore 2010 | UK | Prospective cohort | >18, diagnosis of VLU, ABPI ≥ 0.8, ulcer present > 2 months, ulcer area 2-100cm^2^, no immunosuppressants within 1 month of enrolment, malignant diseases in remission for 3 years or more | 178 participants | 12 weeks | Healed ulcer (assessed visually) at 12 weeks | Logistic regression model | 5 factors:  Bacterial species identified  Taxonomic group  Presence of anaerobes  Identification of Cornyebacterium striatum  Anaerobes identified |
| Mościcka 2023 | Poland | Retrospective cohort (Retrospective notes review from a prospective cohort) | Patients with VLU with APBI between 0.9 and 1.3 and the diagnosis of chronic venous insufficiency confirmed on Duplex scan. Must have a Clinical-Aetiology-Anatomy-Pathophysiology (CEAP) score of C6 | 754 participants | 12 weeks | Ulceration area as a percentage and if completely healed during the study period. | Multivariate ANOVA model | Not reported |
| Parker 2016 | Australia | Secondary analysis of 7 longitudinal prospective studies | Recruited from hospital outpatient clinics and community settings. Leg ulcers of primarily venous aetiology as diagnosed by clinician in charge of care based on clinical signs and symptoms, ABPI and full patient assessment. ABPI between 0.7-1.3 in affected leg. No patients with cognitive impairment and no ulcers with malignancy present (diagnosed by clinician in charge in charge of care) | 318 ulcers in 247 participants | 24 weeks | A healed ulcer at 24 weeks. Healed defined as 100% epithelialisation of the wound bed maintained for at least two weeks as evaluated by the clinician in charge of care and supported with photos. | Generalized linear mixed model | 4 factors:  Living alone  Compression  PUSH score  Ulcer area reduction |
| Pihlaja 2022 | Finland | Retrospective cohort | Patients with VLU, ABPI of 0.8 and higher, ulcer size between 1 and 25 cm and an ulcer duration of 4 years and less. | 273 ulcers in 259 participants | Unclear | Unclear: Delayed ulcer healing was defined as poor healing (slow or no ulcer diameter reduction) after initial endovenous treatment(s), typically 2 to 4 months after the first treatment. | Multivariate logistic regression | 5 factors:  Mobility  Persistent superficial vein reflux after ablation  Recanalization in treated segments  Small saphenous vein Reflux  History of deep vein thrombosis |
| Phillips 2000 | Not specified | Randomised controlled trial (Prospective cohort formed on basis of data from an RCT) | People ≥ 21 years, with one or more venous ulcers larger than 1 cm^2^ in area, a minimum ulcer duration of 2 month, and venous insufficiency confirmed | 165 participants | 12 weeks | 100% healing of the target ulcer, time to healing of the target ulcer, with ulcer area calculated by computerized planimetry | Logistic regression model; Cox modelling | No data reported |
| Rajhathy 2020 | Canada | Retrospective cohort (Secondary analysis of 6 studies: 2 RCTs, 2 cohort studies and 2 pre/post evaluations of evidence-based practice implementation) | Patient with VLU and APBI >0.8 and have documented surface area measurements (cm2) at baseline, 3 months, and 6 months. | 777 participants | 3 and 6 months | Healed or not at 3 months | Multivariate Logistic regression using a backward stepwise regression method | 21 factors:  Mobility  Age on admission  Sex  Family history of leg ulcers  Varicose veins  DVT  Venous surgery  Trauma  Osteoarthritis  Phlebitis  Diabetes  Rheumatoid arthritis  Renal disease  Vasculitis  Ulcerative colitis  Current smoker  Past smoker  Affected leg  Wound area  Anxiety/depression  Feels depressed |
| Skene 1992 | UK | Prospective cohort (Secondary analysis of a single RCT) | Subjects stratified by initial ulcer diameter (greater than 2cm), venous origin, four months maximum duration of follow-up, ABPI >0.75 | 200 participants | 4 months | Time to complete healing of the ulcer. | Cox proportional hazard regression models | 4 factors:  Age  Wound area  Wound duration  Deep vein involvement on photoplethysmography |
| Szewczyk 2009 | Poland | Randomised controlled trial (Prospective cohort formed on basis of data from an RCT) | People older than 18 years with confirmed venous leg ulcer, being treated in a hospital's outpatient venous ulcer clinic | 121 ulcers from 112 participants | 48 weeks | Time to wound healing: defined as complete healing till the restoration of epidermal continuity | Cox proportional hazards model | 2 factors:  Isolated superficial venous insufficiency  Superficial and perforating system insufficiency |
| Taylor 2002 | UK | Retrospective cohort study | Patients who attended Salford primary care trust between January 1997 and December 1999. Ulcer had to be of venous aetiology. | 325 participants with 345 ulcers | 24 weeks | Time to complete ulcer healing. Healing was judged to be complete when all ulcers from a patient had healed. | Cox proportional hazards model | 4 factors:  Age  Wound area  Sex  History of previous leg ulcer |
| Yang 2016 | Canada | Retrospective cohort | People with VLU (diagnosed largely via clinical assessment with further confirmatory and diagnostic tests in some cases) recruited from a regional wound healing clinic over a 3-year period. | 554 participants | 12 months | Number of ulcers healed (in 12 months) | Multivariate binomial analyses | 13 factors:  Age  Sex  Hypertension  Dyslipidemia  Smoking history  Diabetes  History of deep vein thrombosis  Trauma leading to VLU  Obesity  Coronary artery disease  Peripheral arterial disease  Largest ulcer size  Wound duration |

**Table 2. Results of data synthesis for all the 117 factors alongside GRADE assessment results where applicable**

| **Factors** | **Definition** | **Outcome** | **Measures** | **No. of studies** | **No. of participants** | **Effect estimate (95% CI), random effects model** | **Heterogeneity** | **Certainty of evidence** | **References** |
| --- | --- | --- | --- | --- | --- | --- | --- | --- | --- |
| **Patient-level characteristics** | | | | | | | | | |
| Age | Age in years | Time to complete healing (median 24 weeks, range: 16 weeks to 3 years) | HR | 4 | 2202 | 0.99 (0.97 to 1) | tau^2 < 0.0001 [0.0000; 0.0010]; I^2 = 50.0% | Moderate certainty: no downgrading for RoB (200/2202 at high, the remaining moderate), no downgrading for indirectness, no inconsistency, no imprecision, downgraded once for publication bias (under-reporting of one study result); no upgrading | Barwell, 2000; Skene, 1992; Kulkarni, 2007; Gohel 2005 |
| Age | Age in years | Complete healing (median 24 weeks, range: 12 weeks to 52 weeks) | OR | 6 | 2218 | 1.00 (0.99 to 1.02) | tau^2 < 0.0001, I^2 = 22.2% | Moderate certainty: downgraded once for RoB (1006/22182 at high, the remaining moderate), no downgrading for indirectness, no downgrading for inconsistency, no downgrading for imprecision, no downgrading for publication bias; no upgrading | Yang, 2016; Milic, 2009; Karanikolic 2015; Rajhathy, 2020; Margolis, 1999; Cardinal 2009 |
| Age | Categories of age using different cut-offs (3) or results unspecified | NR | Narrative | 4 | 1128 | Inconsistency with non significance (3/4) suggesting no association, but significance (1/4) suggesting better healing outcomes among older people | NR | Very low certainty: downgraded once for RoB (748/1128 at high, the remaining moderate), no downgrading for indirectness, downgraded once for inconsistency, no downgrading for imprecision, downgraded once for publication bias (under-reporting of results); no upgrading | Franks 1995; Taylor, 2002; Aloweni, 2022; Chan 2023 |
| Female | Female | Complete healing (12 weeks, 24 weeks) | HR | 2 | 1902 | 1.16 (0.13 to 10.32) | tau^2 = 0.0515; I^2 = 86.6% | Very low certainty: no downgrading for RoB (all at moderate RoB), no downgrading for indirectness, downgraded once for inconsistency (some heterogeneity), downgraded twice for imprecision (very wide confidence interval), no downgrading for publication bias; no upgrading | Marston, 2017; Gohel 2005 |
| Female | Female | Complete healing (12 weeks, 52 weeks) | OR | 4 | 966 | 0.63 (0.39 to 1.03) | tau^2 = 0; I^2 = 0.0% | Low certainty: no downgrading for RoB (all at moderate RoB), no downgrading for indirectness, no downgrading for inconsistency, downgraded once for imprecision (wide confidence interval), downgraded once for publication bias (under reporting of results); no upgrading | Franks 1995; Milic, 2009; Margolis, 1999; Cardinal 2009 |
| Female | Various definitions or reference unspecified | Complete healing (median 25 weeks, range 12 weeks to 3 years) | Narrative | 4 | 2248 | Consistency, no significance | NR | Very low certainty: downgrading twice for RoB (1464/2248 at high RoB), no downgrading for indirectness, no downgrading for inconsistency, no downgrading for imprecision, downgraded once for publication bias (under-reporting of results); no upgrading | Yang, 2016; Kulkarni, 2007; Karanikolic 2015; Rajhathy, 2020; Barwell, 2000 |
| Diabetes | Diabetes | Complete healing (median 24 weeks, range: 12 weeks to 3 years) | HR | 5 | 3077 | 1.04 (0.96 to 1.12) | tau^2 = 0; I^2 = 0.0% | Low certainty: downgraded once for RoB (842/3077 at high RoB, the remaining at moderate), no downgrading for indirectness, no downgrading for inconsistency, downgraded once for imprecision (wide confidence interval), no downgrading for publication bias; no upgrading | Marston, 2017; Kulkarni, 2007; Gohel 2005; Chan 2023; Barwell, 2000 |
| Diabetes | Diabetes | Complete healing (median 24 weeks, range: 12 weeks to 52 weeks) | OR | 5 | 2029 | 0.99 (0.58 to 1.69) | tau^2 = 0.0609; I^2 = 23.5% | Low certainty: downgraded once for RoB (877/2029 at high RoB, the remaining at moderate), no downgrading for indirectness, no downgrading for inconsistency, downgraded once for imprecision (wide confidence interval), no downgrading for publication bias; no upgrading | Yang, 2016; Rajhathy, 2020; Karanikolic 2015; Margolis, 1999; Cardinal 2009 |
| DVT | History or presence of DVT | Complete healing (median 24 weeks, range: 24 weeks to 27 weeks) | HR | 3 | 1673 | 0.85 (0.41 to 1.76) | tau^2 = 0; I^2 = 63.5% | Low certainty: no downgrading for RoB (255/1673 at high RoB, the remaining at moderate), no downgrading for indirectness, no downgrading for inconsistency, downgraded twice for imprecision (very wide confidence interval), no downgrading for publication bias; no upgrading | Kulkarni, 2007; Gohel 2005; Chan 2023 |
| DVT | History or presence of DVT | Complete healing (median 24 weeks, range: 12 weeks to 52 weeks) | OR | 7 | 2258 | 0.86 (0.67 to 1.11) | tau^2 = 0; I^2 = 0.0% | Low certainty: downgraded once for RoB (1136/2258 at high RoB, the remaining at moderate), no downgrading for indirectness, no downgrading for inconsistency, downgraded once for imprecision (wide confidence interval), no downgrading for publication bias; no upgrading | Yang, 2016; Pihlaja, 2022; Milic, 2009; Rajhathy, 2020; Karanikolic 2015; Margolis, 1999; Cardinal 2009 |
| Obesity | Obesity or BMI>35 | Complete healing (52 weeks) | OR | 2 | 754 | 1.18 (0.09 to 15.44) | I^2 = 0.0%; tau^2 = 0 | Low certainty: no downgrading for RoB (754/754 at moderate RoB), no downgrading for indirectness, no downgrading for inconsistency, downgraded twice for imprecision (very wide confidence interval), no downgrading for publication bias; no upgrading | Yang, 2016; Milic, 2009 |
| Hypertension | Hypertension | Complete healing (median 24 weeks, range: 12 weeks to 52 weeks) | OR | 3 | 1152 | 0.99 (0.43 to 2.28) | tau^2 < 0.0001; I^2 = 0.0% [0.0%; 89.6%] | Low certainty: no downgrading for RoB (1152/1152 at moderate RoB), no downgrading for indirectness, no downgrading for inconsistency, downgraded twice for imprecision (very wide confidence interval), no downgrading for publication bias; no upgrading | Yang, 2016; Margolis, 1999; Cardinal 2009 |
| Coronary artery disease | Various conditions including coronary artery disease (1), myocardial infarction (1), cerebral vascular accident (1), angina (1) | Complete healing (24 weeks and 52 weeks) | Narrative | 2 | 814 | Inconsistency, largely the presence of conditions means poor healing | NR | Very low certainty: no downgrading for RoB (814/814 at moderate RoB), no downgrading for indirectness, downgraded twice for inconsistency, downgraded once for imprecision (small sample size), no downgrading for publication bias; no upgrading | Yang, 2016; Margolis, 1999 |
| Peripheral arterial disease | Peripheral arterial disease | Time to complete healing (6 months) | HR | 1 | 255 | 0.41 (0.13 to 1.27) | NR | Very low certainty: downgraded twice for RoB (255/255 at high RoB), no downgrading for indirectness, no downgrading for inconsistency, downgraded twice for imprecision (very small sample size, wide confidence interval), no downgrading for publication bias; no upgrading | Chan 2023 |
| Peripheral arterial disease | Peripheral arterial disease | Complete healing (median 24 weeks, range: 12 weeks to 52 weeks) | OR | 3 | 1272 | 0.74 (0.31 to 1.75) | tau^2 = 0; I^2 = 0.0% | Low certainty: no downgrading for RoB (1272/1272 at moderate RoB), no downgrading for indirectness, no downgrading for inconsistency, downgraded twice for imprecision (very wide confidence interval), no downgrading for publication bias; no upgrading | Yang, 2016; Aloweni, 2022; Cardinal 2009 |
| Trauma | Trauma | Complete healing (12 weeks, 52 weeks) | OR | 2 | 1331 | 1.03 (0.03 to 38.71) | tau^2 = 0.1043; I^2 = 62.9% | Very low certainty: downgraded once for RoB (777/1331 at high RoB, the remaining at moderate), no downgrading for indirectness, downgraded once for inconsistency, downgraded twice for imprecision (very wide confidence interval), no downgrading for publication bias; no upgrading | Yang, 2016; Rajhathy, 2020 |
| Rheumatoid arthritis | Rheumatoid arthritis | Complete healing (median 24 weeks, range: 24 weeks to 3 years) | HR | 3 | 2007 | 0.98 (0.57 to 1.69) | tau^2 = 0.0188; I^2 = 27.7% | Low certainty: downgraded once for RoB (587/2007 at high RoB, the remaining at moderate), no downgrading for indirectness, no downgrading for inconsistency, downgraded once for imprecision (wide confidence interval), no downgrading for publication bias; no upgrading | Kulkarni, 2007; Gohel 2005; Barwell, 2000 |
| Rheumatoid arthritis | Rheumatoid arthritis | Complete healing (median 24 weeks, range: 24 weeks to 3 years) | OR | 2 | 1115 | 0.95 (0.29 to 3.17) | tau^2 = 0; I^2 = 0.0% | Very low certainty: downgraded twice for RoB (777/1115 at high RoB, the remaining at moderate), no downgrading for indirectness, no downgrading for inconsistency, downgraded twice for imprecision (very wide confidence interval), no downgrading for publication bias; no upgrading | Rajhathy, 2020; Cardinal 2009 |
| Osteoarthritis | Osteoarthritis | Complete healing (12 weeks) | OR | 2 | 1115 | 0.74 (0.46 to 1.20) | tau^2 = 0; I^2 = 0.0% | Very low certainty: downgraded twice for RoB (777/1115 at high RoB, the remaining at moderate), no downgrading for indirectness, no downgrading for inconsistency, downgraded once for imprecision (wide confidence interval), no downgrading for publication bias; no upgrading | Rajhathy, 2020; Cardinal 2009 |
| Smoking | Smoking history | Time to complete healing (6 months) | HR | 1 | 255 | 0.94 (0.41 to 2.16) | NR | Very low certainty: downgraded twice for RoB (255/255 at high RoB), no downgrading for indirectness, no downgrading for inconsistency, downgraded twice for imprecision (small sample size, very wide confidence interval), no downgrading for publication bias; no upgrading | Chan 2023 |
| Smoking | Smoking history | Complete healing (median 12 weeks; range: 12 weeks to 52 weeks) | OR | 2 | 1331 | 1.03 (0.55 to 1.95) | tau^2 = 0; I^2 = 0.0% | Very low certainty: downgraded once for RoB (777/1331 at high RoB, the remaining at moderate), no downgrading for indirectness, no downgrading for inconsistency, downgraded twice for imprecision (very wide confidence interval), no downgrading for publication bias; no upgrading | Yang, 2016; Rajhathy, 2020 |
| Smoking | Current smoker or unspecified | Complete healing (median 12 weeks; range: 12 weeks to 52 weeks) | Narrative | 2 | 1115 | Consistency in non-significance, with one suggesting smoking increased healing but the other suggesting the opposite | NR | Very low certainty: downgraded twice for RoB (777/1115 at high RoB, the remaining at moderate), no downgrading for indirectness, downgraded once for inconsistency, no downgrading for imprecision, no downgrading for publication bias; no upgrading | Cardinal 2009; Rajhathy, 2020 |
| Lipodermatosclerosis | Lipodermatosclerosis | Complete healing (12 and 24 weeks) | OR | 2 | 598 | 1.08 (0.07 to 16.54) | tau^2 = 0; I^2 = 0.0% | Low certainty: no downgrading for RoB (598/598 at moderate RoB), no downgrading for indirectness, no downgrading for inconsistency, downgraded twice for imprecision (small sample size, very wide confidence interval), no downgrading for publication bias; no upgrading | Margolis, 1999; Cardinal 2009 |
| Dermatitis | Dermatitis | Complete healing (12 and 24 weeks) | OR | 2 | 598 | 0.51 (0.50 to 0.52) | tau^2 = 0; I^2 = 0.0% | Moderate certainty: no downgrading for RoB (598/598 at moderate RoB), no downgrading for indirectness, no downgrading for inconsistency, downgraded once for imprecision (small sample size), no downgrading for publication bias; no upgrading | Margolis, 1999; Cardinal 2009 |
| BMI | BMI | Complete healing (or delayed healing), 12 weeks and 24 weeks | OR | 2 | 438 | 1.06 (0.23 to 4.87) | tau^2 = 0.0126; I^2 = 42.2% | Low certainty: no downgrading for RoB (100/438 at high RoB; the remaining at moderate), no downgrading for indirectness, no downgrading for inconsistency, downgraded twice for imprecision (small sample size, very wide confidence interval), no downgrading for publication bias; no upgrading | Karanikolic 2015; Cardinal 2009 |
| Renal disease | Renal disease | Complete healing (12 and 24 weeks) | OR | 2 | 877 | 1.18 (0.27 to 5.04) | tau^2 = 0; I^2 = 0.0% | Very low certainty: downgraded twice for RoB (877/877 at high RoB), no downgrading for indirectness, no downgrading for inconsistency, downgraded twice for imprecision (small sample size, very wide confidence interval), no downgrading for publication bias; no upgrading | Karanikolic 2015; Rajhathy, 2020 |
| PDFS (pain, depression, fatigue, and sleep disturbance) cluster (Severe vs mild as reference) | PDFS (pain, depression, fatigue, and sleep disturbance) cluster (Severe vs mild as reference) | Complete healing | HR | 1 | 247 | 0.68 (0.48 to 0.98) | NR | NR | Finlayson, 2017 |
| Patient admitted for acute hospital stay or emergency department visit (Yes vs no) | Patient admitted for acute hospital stay or emergency department visit (Yes vs no) | Healed wound: Hierarchical definition of a wound considered healed (outlined in Supplementary Table 1) Wound Healing Index used | Unknown | 1 | 26713 VLUs | Unknown | NR | NR | Fife, 2020 |
| Previous operations | Previous operations | Complete ulcer healing (complete epithelialization of the reference limb) | OR | 1 | 200 | 1.38 (0.32 to 5.85) | NR | NR | Milic, 2009 |
| Anxiety/depression | Anxiety/depression | Complete healing | OR | 1 | 777 | 1.034 (0.702 to 1.523) | NR | NR | Rajhathy, 2020 |
| Feels depressed | Feels depressed | Complete healing | OR | 1 | 777 | 0.887 (0.548 to 1.436) | NR | NR | Rajhathy, 2020 |
| Moderate and severe Prognostic Nutritional Index (PNI) | Moderate and severe Prognostic Nutritional Index (PNI) | slower VLU healing at 24 weeks | OR | 1 | 354 | 2.5 (1.4 to 4.6) | NR | NR | Hart 2025 |
| Family history of leg ulcers | Family history of leg ulcers | Complete healing | OR | 1 | 777 | 0.838 (0.537 to 1.307) | NR | NR | Rajhathy, 2020 |
| No limb edema on examination | No limb edema on examination | Complete wound healing | OR | 1 | 260 | 0.7 (0.08 to 6.32) | NR | NR | Margolis, 1999 |
| Dyslipidemia (Present vs absent) | Dyslipidemia (Present vs absent) | Complete healing | OR | 1 | 554 | 1.848 (1.052 to 3.246) | NR | NR | Yang, 2016 |
| Ulcerative colitis | Ulcerative colitis | Complete healing | OR | 1 | 777 | 1.398 (0.184 to 10.639) | NR | NR | Rajhathy, 2020 |
| Thyroid disease | Thyroid disease | Complete wound healing | OR | 1 | 260 | 1.77 (0.37 to 8.51) | NR | NR | Margolis, 1999 |
| Employment status | Employment status (Employed vs retired as reference) | Complete healing | OR | 1 | 168 | 0.89 (0.26 to 3.02) | NR | NR | Franks 1996 |
| Employment status | Employment status (Unemployed vs retired as reference) | Complete healing | OR | 1 | 168 | 4.27 (0.37 to 49.33) | NR | NR | Franks 1997 |
| Employment status | Employment status (Unemployed due to illness vs retired as reference) | Complete healing | OR | 1 | 168 | 0.65 (0.15 to 2.91) | NR | NR | Franks 1998 |
| Employment status | Employment status (Other vs retired as reference) | Complete healing | OR | 1 | 168 | 3.44 (0.24 to 48.52) | NR | NR | Franks 1999 |
| Income | Income (State pension vs State pension+private pension as reference) | Complete healing | OR | 1 | 168 | 0.89 (0.4 to 1.96) | NR | NR | Franks 1999 |
| Income | Income (Work vs State pension +private pension as reference) | Complete healing | OR | 1 | 168 | 0.48 (0.12 to 1.99) | NR | NR | Franks 1999 |
| Income | Income (State + private pension vs State pension +private pension as reference) | Complete healing | OR | 1 | 168 | 0.68 (0.17 to 2.76) | NR | NR | Franks 1999 |
| Income | Income (Other vs State pension +private pension as reference) | Complete healing | OR | 1 | 168 | 0.3 (0.03 to 3.33) | NR | NR | Franks 1999 |
| Housing benefit (Yes vs no) | Housing benefit (Yes vs no) | Complete healing | OR | 1 | 168 | 0.81 (0.4 to 1.65) | NR | NR | Franks 1999 |
| Mobility benefit (Yes vs no) | Mobility benefit (Yes vs no) | Complete healing | OR | 1 | 168 | 0.99 (0.17 to 5.94) | NR | NR | Franks 1999 |
| Housing tenure | Housing tenure (Council tenant vs owner as reference) | Complete healing | OR | 1 | 168 | 0.42 (0.16 to 1.09) | NR | NR | Franks 1999 |
| Housing tenure | Housing tenure (Private tenant vs owner as reference) | Complete healing | OR | 1 | 168 | 0.94 (0.32 to 2.79) | NR | NR | Franks 1999 |
| Housing tenure | Housing tenure (Other vs owner as reference) | Complete healing | OR | 1 | 168 | 0.94 (0.3 to 2.97) | NR | NR | Franks 1999 |
| Satisfaction with housing (No vs Yes) | Satisfaction with housing (No vs Yes) | Complete healing | OR | 1 | 168 | 1.14 (0.42 to 3.12) | NR | NR | Franks 1999 |
| Central heating (No vs Yes) | Central heating (No vs Yes) | Complete healing | OR | 1 | 168 | 2.27 (1.11 to 4.55) | NR | NR | Franks 1999 |
| House occupation (Alone vs with someone as reference) | House occupation (Alone vs with someone as reference) | Complete healing | OR | 1 | 168 | 1.21 (0.6 to 2.44) | NR | NR | Franks 1999 |
| Contact with family/friends | Contact with family/friends (Each week vs each day as reference) | Complete healing | OR | 1 | 168 | 1.84 (0.78 to 4.68) | NR | NR | Franks 1999 |
| Contact with family/friends | Contact with family/friends (Less often vs each day as reference) | Complete healing | OR | 1 | 168 | 5.33 (0.91 to 31.09) | NR | NR | Franks 1999 |
| Living alone (Yes/No) | Living alone (Yes/No) | A healed ulcer at 24 weeks | OR | 1 | 318 VLUS in 247 participants | 2.3 (1.13 to 4.61) | NR | NR | Parker, 2016 |
| Phlebitis | Phlebitis | Complete healing | OR | 1 | 777 | 1.096 (0.6 to 2.004) | NR | NR | Rajhathy, 2020 |
| Vasculitis | Vasculitis | Complete healing | OR | 1 | 777 | 0.832 (0.17 to 4.069) | NR | NR | Rajhathy, 2020 |
| Social class | Social class (III vs I&II as reference) | Complete healing | OR | 1 | 168 | 2.63 (0.83 to 8.3) | NR | NR | Franks 1995 |
| Social class | Social class (IV&V vs I&II as reference) | Complete healing | OR | 1 | 168 | 2.67 (0.81 to 8.85) | NR | NR | Franks 1995 |
| Insurance status as a marker of socioeconomic status (public assistance and self-pay vs those with medical insurance) | Insurance status as a marker of socioeconomic status (public assistance and self-pay vs those with medical insurance) | Complete healing | OR | 1 | 260 | 1.3 (0.38 to 4.48) | NR | NR | Margolis, 1999 |
| Marital status | Marital status (Single vs married as reference) | Complete healing | OR | 1 | 168 | 2.29 (0.86 to 6.08) | NR | NR | Franks 1995 |
| Marital status | Martial status (Widowed vs married as reference) | Complete healing | OR | 1 | 168 | 1.26 (0.52 to 3.08) | NR | NR | Franks 1995 |
| Marital status | Marital status (Divorced/sep vs married as reference) | Complete healing | OR | 1 | 168 | 1.52 (0.36 to 6.39) | NR | NR | Franks 1995 |
| History of previous leg ulcer | History of previous leg ulcer | Time to complete ulcer healing. Healing was judged to be complete when all ulcers from a patient had healed | Unknown | 1 | 325 patients with 345 ulcers | NR | NR | NR | Taylor, 2002 |
| Prior wound history | Prior wound history | incidence of complete wound healing and time to wound healing (complete epithelization of VLU) | HR | 1 | 255 participants | 0.939 (0.415 to 2.12) | NR | NR | Chan 2023 |
| Height (cm) | Height (cm) | Complete healing | OR | 1 | 338 patients | 1.05 (0.93 to 1.19) | NR | NR | Cardinal 2009 |
| Weight (kg) | Weight (kg) | Complete healing | OR | 1 | 338 patients | 0.94 (0.84 to 1.04) | NR | NR | Cardinal 2009 |
| Anemia (yes : no) | Anemia (yes : no) | Complete healing | OR | 1 | 338 patients | 1.72 (0.41 to 7.17) | NR | NR | Cardinal 2009 |
| Stroke | Stroke | Complete healing | OR | 1 | 338 patients | 0.88 (0.19 to 4.05) | NR | NR | Cardinal 2009 |
| Congestive heart failure | Congestive heart failure | Complete healing | OR | 1 | 338 patients | 0.58 (0.16 to 2.18) | NR | NR | Cardinal 2009 |
| Alcohol consumption | Alcohol consumption | Complete healing | OR | 1 | 338 patients | 0.73 (0.37 to 1.43) | NR | NR | Cardinal 2009 |
| Hyperpigmentation | Hyperpigmentation | Complete healing | OR | 1 | 338 patients | 1.57 (0.77 to 3.21) | NR | NR | Cardinal 2009 |
| Race | Non-Chinese vs Chinese | Complete healing | OR | 1 | 380 | 1.01 (0.56 to 1.78) | NR | NR | Aloweni, 2022 |
| Race | Other | incidence of complete wound healing and time to wound healing (complete epithelization of VLU) | HR | 1 | 255 participants | 0.786 (0.367 to 1.68) | NR | NR | Chan 2023 |
| Race | Non-white | Complete wound healing | OR | 1 | 260 | 1.88 (0.8 to 4.45) | NR | NR | Margolis, 1999 |
| Waist/hip ratio | Waist/hip ratio | Delay of healing | OR | 1 | 100 | 3.91 (0.04 to 90.9) | NR | NR | Karanikolic 2015 |
| **Ulcer-level characteristics** | | | | | | | | | |
| Wound duration | Wound duration (months) | Time to complete healing (median 24 weeks, range: 12 weeks to 3 years) | HR | 6 | 3166 | 0.99 (0.98 to 1.00) | tau^2 < 0.0001; I^2 = 85.9% | Low certainty: downgraded once for RoB (1264/3166 at high, the remaining moderate), no downgrading for indirectness, downgraded once for some heterogeneity, no imprecision, no downgrading for publication bias; no upgrading | Marston, 2017; Kulkarni, 2007; Gohel 2005; Chan 2023; Escaleira, 2010; Barwell, 2000 |
| Wound duration | Wound duration (months) | Complete healing (median 24 weeks, range 12 week to 12 months) | OR | 3 | 1152 | 0.88 (0.60 to 1.27) | tau^2 = 0.0160; I^2 = 80.4% | Very low certainty: no downgrading for RoB (all data at moderate), no downgrading for indirectness, downgraded once for some heterogeneity, downgraded twice for the wide confidence interval, no downgrading for publication bias; no upgrading | Yang, 2016; Margolis, 1999; Cardinal 2009 |
| Wound duration | Various definitions or references, ulcer chronicity (1), wound duration in months on a log scale (2), wound duration > 12 months (1), definition or results unspecified (3) | Complete healing (median 16 weeks, range 12 weeks to 3 years) | Narrative | 7 | 57284 | Significance (5/7), non significance or unclear (2/7); all consistently suggesting that shorter wound duration, better healing outcomes | NR | Low certainty: downgraded once for RoB (30,169/57,284 data at high, the remaining at moderate), no downgrading for indirectness, no downgrading for inconsistency, no downgrading for imprecision, downgraded once for publication bias (under-reporting of results); no upgrading | Barwell, 2000; Fife, 2020; Skene, 1992; Milic, 2009; Phillips 2000; Lantis, 2013; Gelfand 2002 |
| Wound area | Size cm2 | Complete healing (median 9 months, range: 12 weeks to 3 years) | HR | 5 | 1979 | 0.96 (0.91 to 1.01) | tau^2 = 0.0017; I^2 = 93.0% | Very low certainty: downgraded twice for RoB (1263/1979 at high RoB, the remaining at moderate), no downgrading for indirectness, no downgrading for inconsistency, no downgrading for imprecision, downgraded once for publication bias (under-reporting of results for two studies); no upgrading | Escaleira, 2010; Lantis, 2013; Chan 2023; Barwell, 2000; Marston, 2017 |
| Wound area | Size cm2 | Complete healing (median 12 weeks, range: 12 weeks to 12 months) | OR | 4 | 1929 | 0.90 (0.76 to 1.08) | tau^2 = 0.0115, I^2 = 93.0% | Very low certainty: no downgrading for RoB (777/1929 at high RoB, the remaining at moderate), no downgrading for indirectness, downgraded once for inconsistency (some heterogeneity), downgraded once for imprecision, downgraded once for publication bias (under-reporting of results for two studies); no upgrading | Yang, 2016; Margolis, 1999; Rajhathy, 2020; Cardinal 2009 |
| Wound area | Various definitions or references, wound area change percentage or <25% at week 2 (2), wound area on a log10 scale (2), wound area >20cm2 (1), unspecified definition or no result (3) | Complete healing (median 24 weeks, range: 12 weeks to 52 weeks) | Narrative | 8 | 84,514 | Significance (4/5), non significance (1/5); all consistently suggesting that smaller areas, better healing outcomes | NR | Moderate certainty: no downgrading for RoB (29,783/84,514 at high RoB, the remaining at moderate), no downgrading for indirectness, no downgrading for inconsistency, no downgrading for imprecision, downgraded once for publication bias (under-reporting of results); no upgrading | Fife, 2020; Skene, 1992; Taylor, 2002; Milic, 2009; Phillips 2000; Parker, 2016; Chaby 2013; Gelfand 2002 |
| Infection | Presence of wound infection | Complete healing (median 24 weeks, range: 12 weeks to 24 weeks) | Narrative | 3 | 27068 | Conflicting results: significance (2/3), suggesting that infection delayed the complete healing of venous leg ulcers; non-significance (1/3) | NR | Very low certainty: no downgrading for RoB (355/27068 at high RoB, the remaining at moderate), no downgrading for indirectness, downgraded twice for inconsistency, no downgrading for imprecision, downgraded once for publication bias (under-reporting of results); no upgrading | Fife, 2020; Karanikolic 2015; Chan 2023 |
| No. of limbs | Two limbs involved | Complete healing (12 weeks, 6 months) | OR | 2 | 548 | 1.08 (0.86 to 1.37) | I^2 = 0.0%; tau^2 = 0 | Low certainty: no downgrading for RoB (168/548 at high RoB, the remaining at moderate), no downgrading for indirectness, no downgrading for inconsistency, downgraded twice for imprecision (small sample size and wide confidence interval), no downgrading for publication bias; no upgrading | Franks 1999; Aloweni, 2022 |
| Wound covered with fibrin>50% | Wound covered with fibrin>50% | Complete healing (24 and 52 weeks) | OR | 3 | 560 | Heterogenous and inconsistent results among the three studies, with one significant result and a non-significant result suggesting that >50% wounds with fibrin delayed healing but the other with non-significance suggesting the opposite | NR | Very low certainty: no downgrading for RoB (100/560 at high RoB, the remaining at moderate), no downgrading for indirectness, downgraded twice for inconsistency, downgraded once for imprecision (small sample size), no downgrading for publication bias; no upgrading | Margolis, 1999; Milic, 2009; Karanikolic 2015 |
| PUSH score | PUSH score (0-17 scale) | Complete healing | HR | 1 | 247 | 0.86 (0.81 to 0.91) | NR | NR | Finlayson, 2017 |
| PUSH score | PUSH score≥ 10 (Yes, No) | A healed ulcer at 24 weeks | OR | 1 | 318 ulcers in 247 participants | 5.1 (2.33 to 11.18) | NR | NR | Parker, 2016 |
| Margolis index score | Margolis index score (1 vs 0) | Time to complete healing | HR | 1 | 802 | 0.48 (0.39 to 0.59) | NR | NR | Jull, 2023 |
| Margolis index score | Margolis index score (2 vs 1) | Time to complete healing | HR | 1 | 802 | 0.3 (0.21 to 0.42) | NR | NR | Jull, 2023 |
| Margolis index score | Margolis index score (2 vs 0) | Time to complete healing | HR | 1 | 802 | 0.14 (0.1 to 0.21) | NR | NR | Jull, 2023 |
| Ulcer location (right or left leg, reference unspecified) | Ulcer location (right or left leg, reference unspecified) | A healed ulcer at 24 weeks | HR | 1 | 231 patients | 0.986 (0.785 to 1.239) | NR | NR | Kulkarni, 2007 |
| Locoregional characteristics of chronic venous ulceration (number of changes) | Locoregional characteristics of chronic venous ulceration (number of changes) | Delay of healing | narrative | 1 | 100 | NR | NR | NR | Karanikolic 2015 |
| Locoregional characteristics of chronic venous ulceration (surface) | Locoregional characteristics of chronic venous ulceration (surface) | Delay of healing | narrative | 1 | 100 | NR | NR | NR | Karanikolic 2015 |
| Locoregional characteristics of chronic venous ulceration (localization) | Locoregional characteristics of chronic venous ulceration (localization) | Delay of healing | narrative | 1 | 100 | NR | NR | NR | Karanikolic 2015 |
| Local complications of chronic venous ulceration (dermatitis) | Local complications of chronic venous ulceration (dermatitis) | Delay of healing | narrative | 1 | 100 | NR | NR | NR | Karanikolic 2015 |
| Local complications of chronic venous ulceration (lipodermosclerosis) | Local complications of chronic venous ulceration (lipodermosclerosis) | Delay of healing | narrative | 1 | 100 | NR | NR | NR | Karanikolic 2015 |
| Local complications of chronic venous ulceration (> 50% of wound covered with eschar) | Local complications of chronic venous ulceration (> 50% of wound covered with eschar) | Delay of healing | narrative | 1 | 100 | NR | NR | NR | Karanikolic 2015 |
| Local complications of chronic venous ulceration (limb edema) | Local complications of chronic venous ulceration (limb edema) | Delay of healing | narrative | 1 | 100 | NR | NR | NR | Karanikolic 2015 |
| Depth of the wound>2cm (Yes/No) | Depth of the wound>2cm (Yes/No) | Complete ulcer healing (complete epithelialization of the reference limb) | OR | 1 | 200 | 1.59 (0.27 to 9.4) | NR | NR | Milic, 2009 |
| Emergence of new skin islets: New islets on more than 10% ulcer surface during first 50 days of treatment (Yes/No) | Emergence of new skin islets: New islets on more than 10% ulcer surface during first 50 days of treatment (Yes/No) | Complete ulcer healing (complete epithelialization of the reference limb) | OR | 1 | 200 | 0.38 (0.04 to 3.43) | NR | NR | Milic, 2009 |
| Right leg (Yes/No) | Right leg (Yes/No) | ulcer healing (complete re-epithelialisation) | HR | 1 | 1186 patients (1324 legs) | 1.04 (0.91 to 1.2) | NR | NR | Gohel 2005 |
| Affected leg | Affected leg | Complete healing | OR | 1 | 777 | 0.891 (0.684 to 1.159) | NR | NR | Rajhathy, 2020 |
| Presence of slough in wound | Presence of slough in wound | incidence of complete wound healing and time to wound healing (complete epithelization of VLU) | HR | 1 | 255 participants | 0.481 (0.226 to 1.02) | NR | NR | Chan 2023 |
| Wound margin undermined | wound margin undermined | Complete wound healing | OR | 1 | 260 | 0.92 (0.31 to 2.73) | NR | NR | Margolis, 1999 |
| >50% of wound covered with eschar | >50% of wound covered with eschar | Complete wound healing | OR | 1 | 260 | 0.23 (0.04 to 1.4) | NR | NR | Margolis, 1999 |
| Number of ulcers or wounds of any type (previous or concurrent) | Number of ulcers or wounds of any type (previous or concurrent) | Healed wound: Hierarchical definition of a wound considered healed (outlined in Supplementary Table 1) Wound Healing Index used | Unknown | 1 | 26713 VLUs | NR | NR | NR | Fife, 2020 |
| No. limb ulcers | No. limb ulcers | Complete healing | OR | 1 | 260 | 1.19 (0.81 to 1.73) | NR | NR | Margolis, 1999 |
| Ulcer position (ankle vs calf as the reference) | Ulcer position (ankle vs calf as the reference) | Complete healing | OR | 1 | 338 patients | 3.22 (1.62 to 6.39) | NR | NR | Cardinal 2009 |
| Swelling (yes : no) | Swelling (yes : no) | Complete healing | OR | 1 | 338 patients | 0.29 (0.13 to 0.6) | NR | NR | Cardinal 2009 |
| Initial wound shape | Initial wound shape | Complete healing | OR | 1 | 338 patients | 1.17 (0.95 to 1.45) | NR | NR | Cardinal 2009 |
| Recurring ulcer | Recurring ulcer | Complete healing | OR | 1 | 338 patients | 1.24 (0.63 to 2.43) | NR | NR | Cardinal 2009 |
| Bilateral ulcer | Bilateral ulcer | Complete healing | OR | 1 | 338 patients | 1.3 (0.52 to 3.3) | NR | NR | Cardinal 2009 |
| Wound exudate (less vs more) | Wound exudate (less vs more) | Complete healing | OR | 1 | 338 patients | 0.66 (0.36 to 1.21) | NR | NR | Cardinal 2009 |
| Necrotic tissue (%) | Necrotic tissue (%) | Complete healing | OR | 1 | 338 patients | 0.98 (0.93 to 1.03) | NR | NR | Cardinal 2009 |
| Pain (less vs more) | Pain (less vs more) | Complete healing | OR | 1 | 338 patients | 0.96 (0.64 to 1.44) | NR | NR | Cardinal 2009 |
| Shape change over time (no change vs concave-convex) | Shape change over time (no change vs concave-convex) | Complete healing | OR | 1 | 338 patients | 4.84 (1.98 to 11.91) | NR | NR | Cardinal 2009 |
| **Characteristics related to calf muscle pump function** | | | | | | | | | |
| Mobility | Various definitions or references, wheelchair/bedbound(n = 1), some problem walking (1), daily walking distance <200m (1), unable to walk 1 block (1), leg mobility vs immobile (1), unspecified (1) | Complete healing (median 18 weeks, range: 12 weeks to 3 years) | Narrative | 6 | 2421 | No significance (4/6); significance (2/6); all favouring the categories of no mobility problem regardless of the definitions used | NR | Very low certainty: downgraded twice for RoB (1623/2421 at high RoB, the remaining at moderate), no downgrading for indirectness, no downgrading for inconsistency, no downgrading for imprecision, downgraded once for publication bias (under-reporting of results); no upgrading | Pihlaja, 2022; Rajhathy, 2020; Milic, 2009; Margolis, 1999; Barwell, 2000; Cardinal 2009 |
| Reduction in calf circumference>3cm reduction during first 50 days of treatment (Yes, No) | Reduction in calf circumference>3cm reduction during first 50 days of treatment (Yes, No) | Complete ulcer healing (complete epithelialization of the reference limb) | OR | 1 | 200 | 0.96 (0.24 to 3.86) | NR | NR | Milic, 2009 |
| Calf:ankle circumeference ratio<1.3 (Yes/No) | Calf:ankle circumeference ratio<1.3 (Yes/No) | Complete ulcer healing (complete epithelialization of the reference limb) | OR | 1 | 200 | 42.31 (8.19 to 218.51) | NR | NR | Milic, 2009 |
| Fixed ankle joint ROM <20 degrees (Yes/No) | Fixed ankle joint ROM <20 degrees (Yes/No) | Complete ulcer healing (complete epithelialization of the reference limb) | OR | 1 | 200 | 60.09 (10.8 to 334.44) | NR | NR | Milic, 2009 |
| **Vascular-related characteristics or related pathophysiology variables** | | | | | | | | | |
| ABPI | ABPI<0.8 | Complete healing (24 weeks) | OR | 2 | 360 | Inconsistency and conflicting results, with one suggesting ABPI<0.8 delayed healing but the other suggesting ABPI<0.8 predicted healing | NR | Very low certainty: no downgrading for RoB (100/360 at high RoB, the remaining at moderate), no downgrading for indirectness, downgraded twice for inconsistency, downgraded once for imprecision (small sample sizes), no downgrading for publication bias; no upgrading | Karanikolic 2015; Margolis, 1999 |
| ABPI | ABPI values | Complete healing (median 24 weeks; range 12 weeks to 24 weeks) | OR | 3 | 698 | 0.38 (0.04 to 3.50) | tau^2 = 0; I^2 = 0.0% | Low certainty: no downgrading for RoB (100/698 at high RoB, the remaining at moderate), no downgrading for indirectness, no downgrading for inconsistency, downgraded twice for imprecision (very wide confidence interval), no downgrading for publication bias; no upgrading | Karanikolic 2015; Margolis, 1999; Cardinal 2009 |
| Small saphenous vein reflux | Small saphenous vein reflux | Complete healing (24 weeks) | Narrative | 2 | 514 | Consistency, no significance, with one favouring the factor but the other not | NR | Very low certainty: downgraded twice for RoB (514/514 at high RoB), no downgrading for indirectness, no downgrading for inconsistency, downgraded twice for imprecision (very small sample size), no downgrading for publication bias; no upgrading | Pihlaja, 2022; Chan 2023 |
| Segmental deep reflux | Segmental deep reflux | Complete healing (24 and 30 weeks) | HR | 2 | 1421 | 1.01 (0.69 to 1.47) | tau^2 = 0; I^2 = 0.0% | Low certainty: no downgrading for RoB (all at moderate RoB), no downgrading for indirectness, no downgrading for inconsistency, downgraded twice for imprecision (very wide confidence interval), no downgrading for publication bias; no upgrading | Kulkarni, 2007; Gohel 2005 |
| Total deep reflux | Total deep reflux | Complete healing (24 weeks) | HR | 2 | 1422 | 0.89 (0.75 to 1.05) | tau^2 = 0; I^2 = 0.0% | Moderate certainty: no downgrading for RoB (all at moderate RoB), no downgrading for indirectness, no downgrading for inconsistency, downgraded once for imprecision (wide confidence interval), no downgrading for publication bias; no upgrading | Kulkarni, 2007; Gohel 2005 |
| Superficial venous reflux | Superficial venous reflux | Complete healing (24 weeks and 3 years) | HR | 2 | 1773 | 1.14 (0.85 to 1.53) | tau^2 = 0; I^2 = 0.0% | Very low certainty: downgraded once for RoB (587/1773 at high RoB, the remaining at moderate), no downgrading for indirectness, no downgrading for inconsistency, downgraded twice for imprecision (very wide confidence interval), no downgrading for publication bias; no upgrading | Barwell, 2000; Gohel 2005 |
| Superficial venous reflux | Superficial venous reflux | Complete healing (unspecified timepoint) | OR | 1 | 259 | 0.12 (0.01 to 0.30) | NR | Very low certainty: downgraded twice for RoB (259/259 at high RoB), no downgrading for indirectness, no downgrading for inconsistency, downgraded twice for imprecision (very small sample size), no downgrading for publication bias; no upgrading due to very small sample sizes | Pihlaja, 2022 |
| Incompetent perforators | Incompetent perforators | Complete healing (24 weeks) | HR | 2 | 1423 | 1.04 (0.48 to 2.28) | tau^2 = 0; I^2 = 0.0% | Low certainty: no downgrading for RoB (all at moderate RoB), no downgrading for indirectness, no downgrading for inconsistency, downgraded twice for imprecision (very wide confidence interval), no downgrading for publication bias; no upgrading | Kulkarni, 2007; Gohel 2005 |
| Varicose veins | Varicose veins | Complete healing (median 12 weeks; range 12 to 24 weeks) | OR | 3 | 1375 | 1.16 (0.41 to 3.28) | tau^2 = 0.1182; I^2 = 37.6% | Very low certainty: downgraded once for RoB (777/1375 at high RoB, the remaining at moderate), no downgrading for indirectness, downgraded once for inconsistency, downgraded twice for imprecision (very wide confidence interval), no downgrading for publication bias; no upgrading | Cardinal 2009; Margolis, 1999; Rajhathy, 2020 |
| Venous refill time<=20 seconds (Yes/No) | Venous refill time<=20 seconds (Yes/No) | A healed ulcer at 24 weeks | HR | 1 | 239 patients | 0.656 (0.517 to 0.833) | NR | NR | Kulkarni, 2007 |
| Great saphenous vein reflux | Great saphenous vein reflux | incidence of complete wound healing and time to wound healing (complete epithelization of VLU) | HR | 1 | 255 participants | 0.824 (0.339 to 2) | NR | NR | Chan 2023 |
| Localisation of venous pathology: Isolated superficial venous insufficiency (Yes, No) | Localisation of venous pathology: Isolated superficial venous insufficiency (Yes, No) | Complete healing till the restoration of epidermal continuity; time to healing used in Cox regression | narrative | 1 | 112 people (121 ulcers) | | NR | NR | Szewczyk 2009 |
| Venous flow index | Venous flow index | Complete wound healing | OR | 1 | 260 | 0.93 (0.82 to 1.05) | NR | NR | Margolis, 1999 |
| Mixed vein reflux | Mixed vein reflux | Complete healing | HR | 1 | 633 limbs from 587 patients | 0.9 (0.6 to 1.37) | NR | NR | Barwell, 2000 |
| Popliteal vein reflux | Popliteal vein reflux | Complete healing | HR | 1 | 633 limbs from 587 patients | 0.73 (0.6 to 0.91) | NR | NR | Barwell, 2000 |
| Deep vein reflux | Deep vein reflux | Complete healing | HR | 1 | 633 limbs from 587 patients | 0.74 (0.43 to 1.28) | NR | NR | Barwell, 2000 |
| **Medical treatment-related variables** | | | | | | | | | |
| Veins surgery | Veins surgery | Complete healing (median 24 weeks, range 24 weeks to 3 years) | HR | 3 | 2011 | 0.99 (0.80 to 1.23) | tau^2 = 0; I^2 = 0.0% | Moderate certainty: no downgrading for RoB (587/2011 at high RoB, the remaining at moderate), no downgrading for indirectness, no downgrading for inconsistency, downgraded once for imprecision (wide confidence interval), no downgrading for publication bias; no upgrading | Kulkarni, 2007; Gohel 2005; Barwell, 2000 |
| Veins surgery | Veins surgery | Complete healing (median 24 weeks, range 12 weeks to 24 weeks) | OR | 3 | 1141 | 1.11 (0.01 to 131.05) | tau^2 = 3.2571; I^2 = 86.7% | Very low certainty: downgraded twice for RoB (881/1141 at high RoB, the remaining at moderate), no downgrading for indirectness, downgraded twice for inconsistency (very large heterogeneity), downgraded twice for imprecision (very wide confidence interval), no downgrading for publication bias; no upgrading | Rajhathy, 2020; Chaby 2013; Margolis, 1999 |
| Debridement | Debridement | Complete healing (median 24 weeks; range 24 weeks to 52 weeks) | OR | 3 | 840 | 0.44 (0.17 to 1.10) | tau^2 = 0; I^2 = 0.0% | Low certainty: no downgrading for RoB (840/840 at moderate RoB), no downgrading for indirectness, no downgrading for inconsistency, downgraded twice for imprecision (very wide confidence interval), no downgrading for publication bias; no upgrading | Aloweni, 2022; Milic, 2009; Margolis, 1999 |
| Compression | Various definitions including compression pressure (2), compression duration in days (1) | Complete healing (24 weeks) | Narrative | 3 | 874 | All significance; with consistency in suggesting compression improved healing | NR | Moderate certainty: no downgrading for RoB (874/874 at moderate RoB), no downgrading for indirectness, no downgrading for inconsistency, downgraded once for imprecision (small sample size), no downgrading for publication bias; no upgrading | Finlayson, 2017; Aloweni, 2022; Parker, 2016 |
| Hip or knee replacement surgery (Yes or no) | Hip or knee replacement surgery (Yes or no) | Complete wound healing | OR | 1 | 260 | 3.52 (1.12 to 11.08) | NR | NR | Margolis, 1999 |
| Recanalization in treated segments (Yes vs no as reference) | Recanalization in treated segments (Yes vs no as reference) | Complete healing | OR | 1 | 274 ulcers in 259 patients | 0.471 (0.135 to 1.646) | NR | NR | Pihlaja, 2022 |
| Deep vein involvement on photoplethysmography (Yes vs no) | Deep vein involvement on photoplethysmography (Yes vs no) | Time to complete healing of the ulcer | RR | 1 | 203 patients | NR | NR | NR | Skene, 1992 |
| **Biomarkers and microbiology-related factors** | | | | | | | | | |
| Bacterial species identified: no. of specied | Bacterial species identified: no. of specied | Healing at 12 weeks | | 1 | 178 | NR | NR | NR | Moore, 2010 |
| Taxonomic group: over four species | Taxonomic group: over four species | Healing at 12 weeks | | 1 | 178 | NR | NR | NR | Moore, 2010 |
| Presence of anaerobes | Presence of anaerobes | Healing at 12 weeks | | 1 | 178 | NR | NR | NR | Moore, 2010 |
| Identification of Cornyebacterium striatum | Identification of Cornyebacterium striatum | Healing at 12 weeks | | 1 | 178 | NR | NR | NR | Moore, 2010 |
| Anaerobes identified: no. of species | Anaerobes identified: no. of species | Healing at 12 weeks | | 1 | 178 | NR | NR | NR | Moore, 2010 |
| **Surrogate indicators of healing** | | | | | | | | | |
| Percent healing and ulcer area at week 3 | Percent healing and ulcer area at week 3 | 100% healing of the target ulcer, time to healing of the target ulcer | narrative | 1 | 165 | NR | NR | NR | Phillips 2000 |
